# Integrating Spatial Proteogenomics in Cancer Research

**DOI:** 10.1002/advs.202520744

**Published:** 2026-02-08

**Authors:** Yida Wang, Yang Wu, Feng Zhang, Parthiban Periasamy, Haiyue You, Denise Goh, Rachel Elizabeth Ann Fincham, Xin Ning, Danping Wu, Lu Liu, Ying Jiang, Zhiwen Qian, Joe Yeong, Yan Zhang

**Affiliations:** ^1^ Department of Oncology Wuxi Medical Center Wuxi Maternal and Child Health Care Hospital Nanjing Medical University Wuxi China; ^2^ Institute of Molecular and Cell Biology (IMCB) Agency for Science Technology and Research (A*STAR) Singapore Singapore; ^3^ Department of Oncology Wuxi Maternity and Child Health Care Hospital Women's Hospital of Jiangnan University Jiangnan University Wuxi China; ^4^ Department of Anatomical Pathology Singapore General Hospital (SGH) Singapore Singapore

**Keywords:** AI, dark proteome, deep visual proteomics, spatial proteogenomics, tumor microenvironment

## Abstract

**Background**: Spatial proteogenomics marks a paradigm shift in oncology by integrating molecular analysis with spatial information from both spatial proteomics and other data modalities (e.g., spatial transcriptomics), thereby unveiling tumor heterogeneity and dynamic changes in the microenvironment.

**Methods**: We systematically reviewed the evolution of spatial proteogenomics, from single‐modality profiling to integration with transcriptomics and metabolomics, from the detection of abundant proteins to exploration of “dark proteome” with low abundance or stability, and from analytic software based on traditional machine learning algorithms to advanced artificial intelligence–driven analytical frameworks.

**Results**: Key advances of sub‐fields of spatial proteogenomics include:

- RNA‐protein co‐localization: Spatial CITE‐seq, enabling RNA–protein co‐localization to reveal immune microenvironmental patterns and neoantigen distribution.
- Spatial Proteomics + Spatial Metabolomics: Matrix‐assisted laser desorption/ionization imaging (MALDI), overcoming protein detection bottlenecks and capturing metabolic reprogramming.
- Deep visual proteomics (DVP): achieving unbiased spatial analysis via AI‐guided microdissection.
- Spatial‐aware multiplex dark proteome approaches: Examples are nanodroplet processing in one pot for trace samples (NanoPOTS) and proteoform imaging mass spectrometry (PiMS).
- Multimodal foundation AI models: Examples are KRONOS and HEIST, which integrate multiple data modalities and significantly improve diagnostic precision and therapeutic prediction.

**Conclusions and Future Directions**: Despite challenges of resolution, standardization, and data complexity, spatial proteomics is advancing rapidly. Together with frontier technologies such as quantum computing, live imaging, and organoid integration, it is driving breakthroughs in cancer diagnosis, personalized immunotherapy, and drug development.

## Graphical abstract

Xx xx.

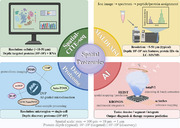

## 1 Introduction

### 1.1 Spatial Proteomic Addresses Protein‐Protein Interaction‐Related Biological Questions

Traditional biological methods have long provided critical insights into cancer biology, with their trajectory having begun from bulk tissue analyses that identified macroscopic molecular trends across cancer populations. With continuous technological advances, the advent of single‐ cell sequencing fundamentally transformed this understanding by revealing pronounced cellular heterogeneity within tumors. However, both approaches have inherent limitations: they obscure local interactions in the tumor microenvironment by averaging cellular signals, while neglecting essential spatial context information [1]. To address these shortcomings, spatial proteomics [2] has emerged as a new evolutionary stage in cancer research, enabling the direct in situ mapping of protein distributions. This technology achieves unprecedented spatial resolution, delineating complex details such as tissue architecture, intercellular communication, immune signaling pathways, and tumor‐specific antigen presentation—marking a transformative era for cancer research and precision medicine. Recognized as the “Method of the Year 2024” [3], spatial proteomics [3] underscores its potential by filling the gaps left by conventional methods and opening vast possibilities for cancer research and therapy.

### 1.2 From Spatial Proteomics to Spatial Proteogenomics

Spatial proteomics reveals the localization of proteins within the tissue microenvironment. However, using spatial proteomics alone cannot address many biological and clinical questions because both its coverage (i.e., the proportion of detected protein molecules relative to the total) and detection depth (i.e., the diversity of molecular species detected) remain constrained by current technologies. On the other hand, while spatial (epi)genomics (spatially characterizing genetic mutations and epigenetic modifications) and transcriptomics (capturing gene expression profiles) [4] have revolutionized our understanding of tissue organization [5], DNA/epigenetic level modifications and RNA abundance do not always faithfully reflect protein abundance, activity, or post‐translational regulation [6]. Thus, protein‐level information is indispensable for capturing signaling states, enzymatic activity, receptor–ligand interactions, and immune effector functions that directly drive tumor behavior [7].

**FIGURE 1:**
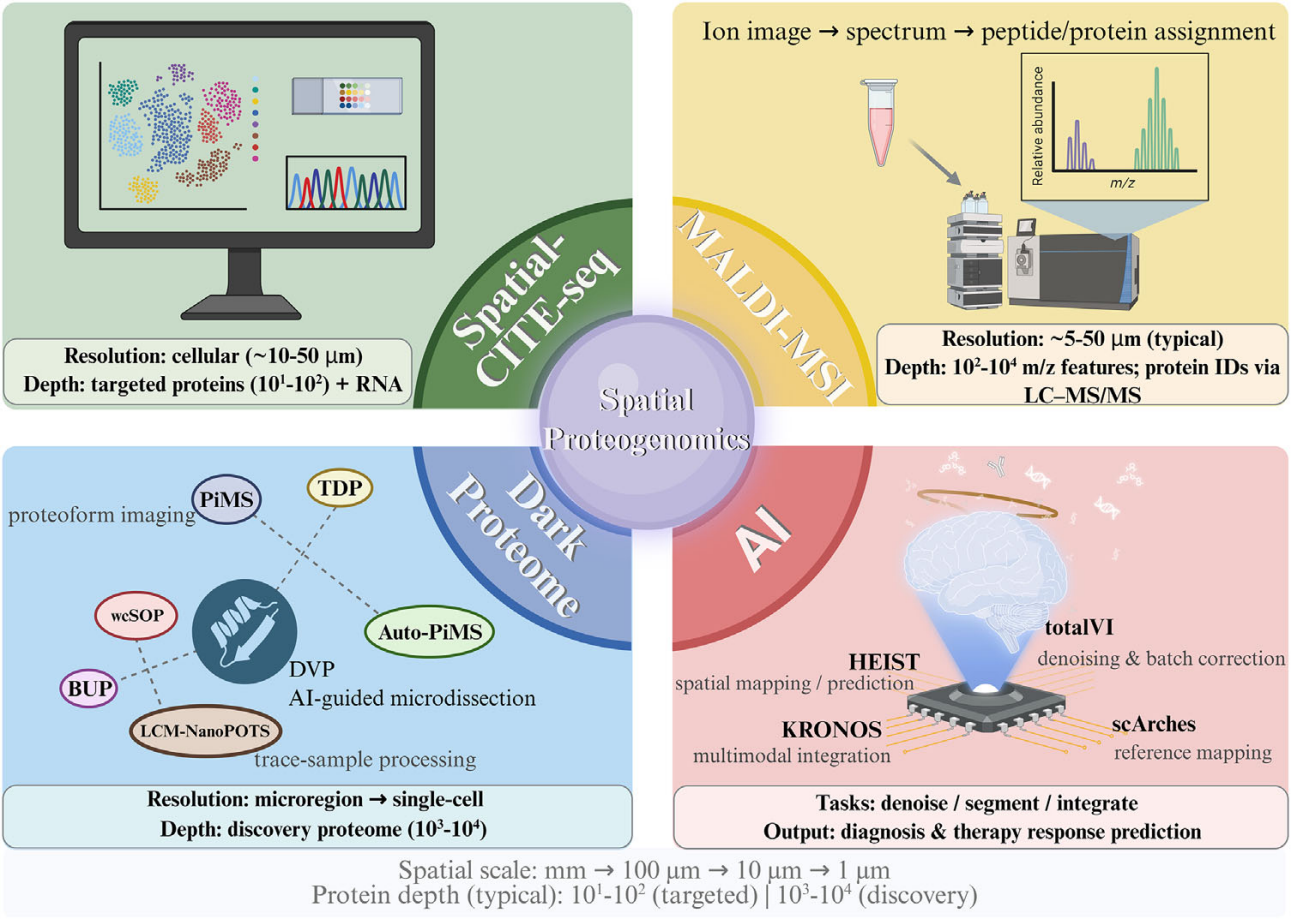
| Overview of key technologies shaping spatial proteogenomics. Spatial proteogenomics integrates molecular profiling with spatial context to resolve intratumoral heterogeneity and dynamic microenvironmental programs. Shown are four representative pillars: (1) RNA‐protein co‐localization: one of the representative platforms is Spatial CITE‐seq, enabling co‐mapping of RNA and targeted proteins (ADTs) at cellular‐scale resolution; (2) Spatial proteomics + metabolomics: MALDI–MSI, providing label‐free ion imaging and spectral readouts that support metabolite/lipid mapping and peptide/protein identification via downstream LC–MS/MS; (3) DVP/dark‐proteome workflows, which combine AI‐guided microdissection with ultrasensitive bottom‐up (e.g., NanoPOTS, wcSOP) and top‐down platforms (e.g., PiMS, Auto‐PiMS) to access low‐abundance, noncanonical, or structurally labile proteoforms; and (4) AI foundation models (KRONOS, HEIST, totalVI, scArches) for denoising, reference mapping, multimodal integration, and clinically oriented prediction. The bottom annotations summarize typical spatial scales (mm–µm) and order‐of‐magnitude protein depth across targeted and discovery modes.

**FIGURE 2:**
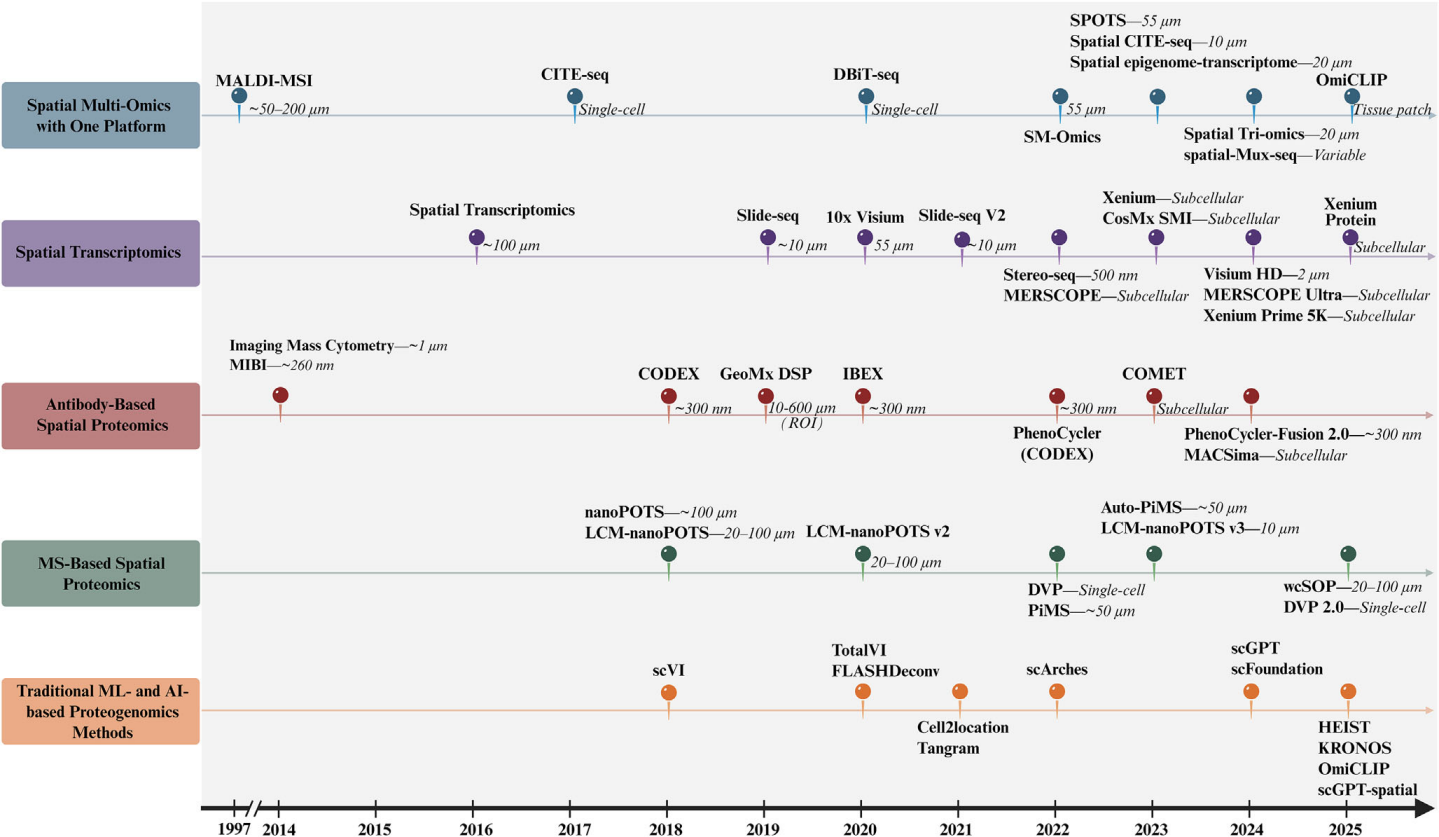
| Evolutionary timeline of spatial proteogenomic technologies (1997–2025). A shared time axis summarizes representative advances across five categories: spatial multi‐omics on a single platform, spatial transcriptomics, antibody‐based spatial proteomics, MS‐based spatial proteomics, and traditional ML/AI‐based proteogenomics methods. Each dot indicates the year a technology was introduced, with labels denoting the platform name; italic annotations report the typical spatial resolution (µm/nm, single‐cell, subcellular, ROI, variable, or tissue patch) as compiled in Table S1. Overall, the timeline illustrates the rapid expansion from early spatial molecular mapping toward higher‐resolution assays and increasingly integrated multimodal profiling, alongside emerging AI‐enabled approaches for data integration and prediction.

To address the limitations of single omics, researchers have turned to “spatial proteogenomics” — integration with complementary spatial omics approaches including spatial transcriptomics, spatial (epi)genomics, spatial metabolomics (profiling small‐molecule metabolites), and other multi‐omics datasets [2, 8] (established technological platforms highlighted in main text are illustrated in Figure 1 and Table 1). This powerful integration strategy is driving cancer research forward with unprecedented momentum [9], leading to breakthroughs in critical areas including targeted therapy (precisely exploiting tumor vulnerabilities), immunotherapy (activating or enhancing the patient's own immune system), and neoantigen discovery for cancer vaccines (identifying individualized targets for immunotherapy) [10, 11]. Spatial proteogenomics therefore represents a necessary evolution (Figure 2, Table S1) rather than an alternative approach, integrating spatial proteomics with transcriptomic and genomic layers to bridge the gap between genetic potential and functional execution [12].

**TABLE 1:** Established Technological Platforms in Spatial Proteogenomics: Principles, Strengths, Limitations, and Mitigation Strategies.

Platform/Technology	Principle	Key advantages	Limitations	Potential solutions
RNA‐protein co‐localizatoin: Spatial CITE‐seq	Integrates spatial transcriptomic and proteomic readouts on a single tissue section via barcoded surface‐capture and antibody‐derived tags (ADTs) to map RNA and protein distributions concurrently	Joint RNA–protein capture Preserves spatial context Resolves cell–cell communication Dissects TME heterogeneity	Throughput constrained by antibody availability Limited spatial resolution High analytical complexity Lack of standardized workflows	Develop next‐gen multiplex labeling Integrate super‐resolution imaging Establish unified technical standards Build efficient computational pipelines
MALDI‐Based Spatial Metabolomics (MALDI‐MSI)	Matrix‐assisted laser desorption/ionization of matrix‐coated tissue enables in situ visualization of metabolites/lipids by m/z	Label‐free detection High sensitivity to small molecules Reveals metabolic rewiring Directly reflects functional states	Poor performance for proteins Low ionization efficiency Matrix‐related interference Challenging for high‐mass species	Optimize nanoparticle matrices On‐tissue enzymatic digestion Complement with antibody‐based imaging Build multimodal integration platforms
Deep Visual Proteomics (DVP/scDVP)	Combines high‐resolution imaging, AI‐guided cell segmentation, laser microdissection, and ultra‐sensitive mass spectrometry to profile phenotypically matched cells	Single‐cell depth (∼1700 zproteins/cell) Maintains spatial context Unbiased proteome coverage Tracks disease progression	Relatively low throughput Specialized instrumentation Complex pre‐analytics High cost	End‐to‐end automation High‐throughput workflows Simplified sample preparation Cost‐reduction of platforms
Dark Proteome Profiling	NanoPOTS/LCM‐NanoPOTS/wcSOP nano‐scale processing with bottom‐up/top‐down proteomics to detect non‐canonical, low‐abundance, or unstable proteins	Captures proteins missed by conventional methods Identifies novel tumor antigens Reveals cryptic therapeutic targets Expands immunotherapy target space	Extremely low abundance Antibody scarcity Instability/degradation Detection threshold limits	Inhibit NMD to boost expression Ultra‐sensitive detection chemistries Optimized one‐pot workflows Integrate TDP for direct analysis
AI‐Driven Integration	Deep learning (e.g., KRONOS, HEIST), variational models (totalVI), and architectural transfer (scArches) for multimodal integration, pattern discovery, and prediction	Automated analytics Cross‐modal fusion Clinical outcome prediction Batch‐effect mitigation	High compute demand Limited interpretability Need for large training cohorts Cross‐platform generalizability	Lightweight, efficient models Interpretable AI methods Shared, curated repositories Federated learning frameworks

It is important to clarify what distinguishes “spatial proteogenomics” from the simple combination of spatial proteomics and spatial transcriptomics or other omics data modalities. The term “spatial proteogenomics” encompasses three distinct but complementary paradigms:

(1) **simultaneous measurement (“Spatial Multi‐Omics in One Platform” in** Table S1), where RNA and protein signals are captured from the same tissue section using unified platforms such as spatial CITE‐seq [13], DBiT‐seq [14], or SPOTS [15]; (2) **computational integration**, where separately acquired spatial proteomics and transcriptomics datasets are aligned through image registration, coordinate mapping, and statistical harmonization methods [16, 17]; and (3) **functional inference**, where protein‐level information is computationally predicted from spatial transcriptomic data using machine learning models trained on matched RNA–protein datasets, or conversely, where transcript abundance is inferred from proteomic measurements [18]. Each paradigm offers unique advantages and limitations (detailed discussion in Section 6.2): simultaneous measurement eliminates registration errors but is constrained by platform‐specific limitations; computational integration enables retrospective analysis of existing datasets but requires careful handling of batch effects and spatial alignment; functional inference extends the utility of single‐modality data but depends on the accuracy of predictive models and the biological validity of RNA–protein correlations [6]. The choice among these approaches should be guided by the specific biological question, available tissue resources, and the required depth of molecular coverage.

## 2 Spatial Multi‐Omics With One Platform: RNA‐Protein or Protein‐Metabolite Co‐Localization

The most intuitive way to integrate multiple omics into spatial proteogenomics, without relying on traditional machine‐learning (ML) or AI‐driven sophisticated image registration and data alignment, is to directly obtain multiple omics data from a single tissue sample using the intrinsic capabilities of one technological platform (hereinafter we termed as “Spatial Multi‐omics with One Platform” or “Spatial Multi‐omics” in short). In this chapter, we introduce two subcategories: (1) RNA‐Protein co‐localization, exemplified by next‐generation sequencing (NGS)‐based methods such as Spatial CITE‐seq [13], DBiT‐seq [14], and SPOTS, as well as commercial platforms like NanoString GeoMx DSP [19]; and (2) Proteome‐lipidome‐metabolome co‐localization, MALDI [20] is one of the most representative examples.

### 2.1 Spatial‐CITE‐seq: Co‐localization of RNA and Protein

**FIGURE 3:**
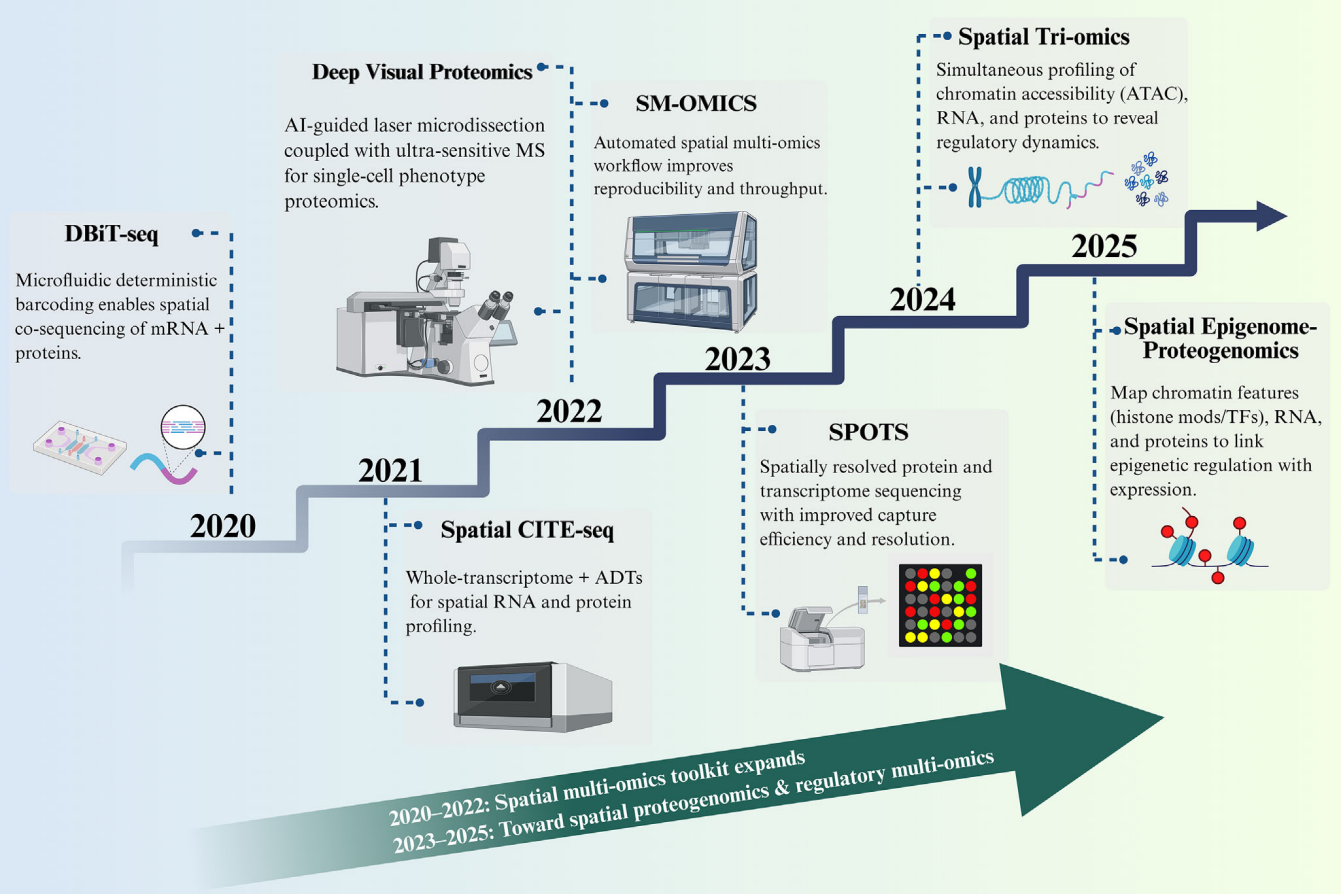
| Milestones in spatial multi‐omics enabling spatial proteogenomics (2020–2025). Stair‐step timeline highlighting major advances that progressively expanded spatial multi‐omics. DBiT‐seq [14] (2020) enabled microfluidic barcoding‐based co‐sequencing of mRNA and proteins. Spatial CITE‐seq [13] (2021) combined whole‐transcriptome profiling with antibody‐derived tags (ADTs) for joint RNA–protein mapping. Deep Visual Proteomics (2022) integrated AI‐guided laser microdissection with ultra‐sensitive mass spectrometry for single‐cell phenotype proteomics, while SM‐OMICS streamlined automated spatial multi‐omics workflows. SPOTS (2023) improved capture efficiency and spatial resolution. Spatial Tri‐omics (2024) co‐profiled ATAC, RNA, and proteins to interrogate regulatory dynamics, and emerging spatial epigenome–proteogenomics (2025) links chromatin features (e.g., histone marks/TFs) with RNA and proteins in situ. The diagonal arrow denotes the shift from toolkit expansion (2020–2022) to regulatory multi‐omics and spatial proteogenomics (2023–2025).

High‐throughput spatial‐CITE‐seq technology [21] ingeniously combines barcoded surface–based spatial transcriptomics with antibody‐derived tag (ADT)–mediated epitope indexing. This approach enables simultaneous spatially resolved profiling of a large panel of proteins (often dozens to hundreds) alongside comprehensive transcriptome information (tens of thousands of expressed genes) from a single tissue slice [13]. It is noteworthy that the advancement of spatial‐CITE‐seq and other state‐of‐the‐art (SOTA) spatial proteogenomics builds upon the rapid evolution of spatial multi‐omics technologies. As early as 2020, DBiT‐seq (Deterministic Barcoding in Tissue), developed by Yang Liu et al. [14]., pioneered the simultaneous high‐spatial‐resolution sequencing of the transcriptome and proteome on the same tissue section. This milestone work was highlighted by Nature Methods as the “Method of the Year,” [22] marking the entry of multi‐omics sequencing into the spatial dimension. Subsequently, SM‐OMICS (2022) [23] further optimized automated workflows, while SPOTS (2023) [15] significantly improved capture efficiency and resolution. More recently, studies have demonstrated the capability to simultaneously detect five modalities—including DNA, RNA, proteins, and metabolites—on a single tissue section [24, 25]. The iteration of these **Spatial Multi‐omics with One Platform** technologies (see Figure 3 and “**Spatial Multi‐omics with One Platform**” section in Table S1 for a detailed timeline) has not only enriched the technological toolkit but also provided a broader perspective for deciphering the tumor microenvironment.

The power of spatial‐CITE‐seq has been vividly demonstrated in studies of mRNA vaccines against SARS‐CoV‐2. When applied to skin biopsy samples from vaccinated individuals, spatial‐CITE‐seq delineated distinctive spatial patterns of immune cell localization at the injection site, together with early activation events such as upregulation of signaling pathways and cytokine secretion [13]. These high‐resolution spatial dynamics provided direct experimental evidence for understanding mechanisms to initiate local immune responses and eventually drive systemic immune responses. Such insights not only advance the mechanistic understanding of vaccine action but also open promising avenues for optimizing vaccine delivery, designing more effective adjuvants, and developing personalized immunological interventions [26].

Similarly, within the tumor immune microenvironment (TIME), spatial proteogenomics enables precise mapping of immune checkpoint molecules (such as PD‐1, PD‐L1, and CTLA‐4) and their ligands at defined spatial locations, while concurrently linking neighboring tumor and immune cells with their gene expression profiles and potential mutational backgrounds [27]. This multidimensional spatial resolution empowers researchers to clearly identify mechanisms underpinning the establishment of immunosuppressive niches—for example, spatial patterns of T cell exhaustion in specific regions and their associations with heterogeneous tumor clones or metabolic states [28].

**FIGURE 4:**
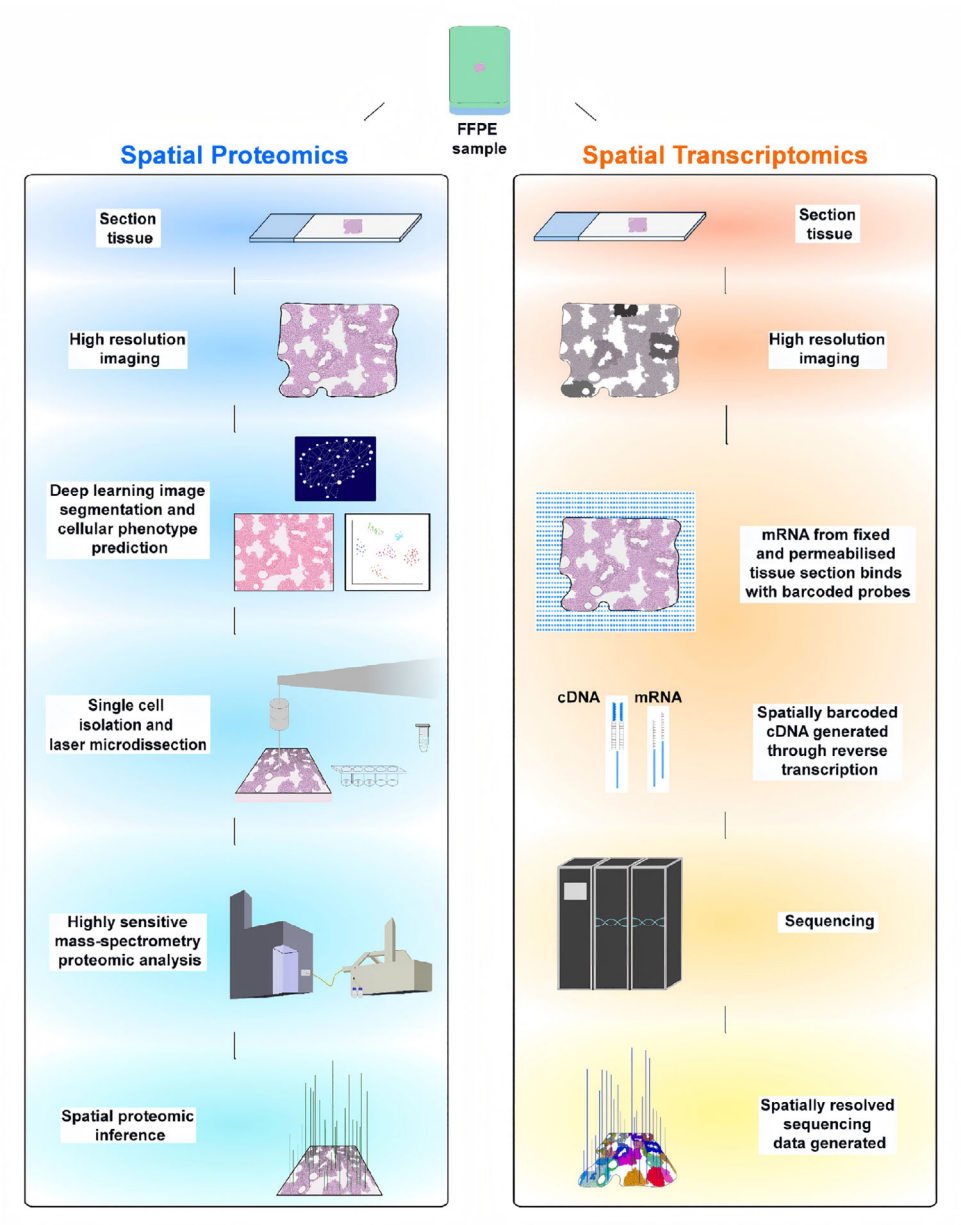
Integrating spatial proteomic workflow and spatial transcriptomic workflow for FFPE tissue sections. Formalin‐fixed paraffin‐embedded (FFPE) tissues are sectioned and processed using parallel spatial proteomic and transcriptomic workflows to achieve high‐resolution, multi‐omic tissue mapping. Spatial proteomics combines deep learning‐driven image segmentation with laser microdissection and mass spectrometry to quantify and spatially infer protein expression at the single‐cell level. In parallel, spatial transcriptomics captures mRNA from permeabilized tissue sections using spatially barcoded probes, enabling cDNA synthesis and sequencing‐based reconstruction of spatial gene expression patterns. Together, these approaches offer a powerful and complementary view of the spatial proteogenomic landscape, revealing cellular phenotypes and molecular heterogeneity within complex tissue architecture.

More importantly, by integrating spatial‐proteomic, transcriptomic, and genomic data, researchers can accurately localize and quantify the spatial distribution patterns of tumor neoantigens, as well as their spatial proximity to infiltrating T cells, within one tissue section. This provides robust spatial evidence for selecting highly immunogenic neoantigens with effective presentation potential, thereby greatly accelerating the development of personalized cancer vaccines [29]. In addition, the technology can uncover functionally distinct spatial niches within tumors—such as highly proliferative regions, invasive margins, immune‐excluded regions, or immune‐enriched (“hot”) regions—and characterize their unique dynamics in their protein expression profiles, gene expression signatures, metabolic reprogramming activities, and cell‐cell communication networks [30]. The integrated spatial proteogenomic workflow is illustrated in Figure 4, which depicts the parallel processing of FFPE tissue sections through both spatial proteomic and transcriptomic pipelines. On the proteomic side, deep learning‐driven image segmentation guides laser microdissection, followed by highly sensitive mass spectrometry analysis to generate spatially resolved protein quantification. Concurrently, the transcriptomic workflow captures mRNA through spatially barcoded probes, enabling cDNA synthesis and sequencing‐based gene expression mapping. This dual‐pathway approach converges to provide a comprehensive view of the spatial proteogenomic landscape.

This unprecedentedly fine‐grained dissection of tumor spatial heterogeneity not only deepens our understanding to tumor initiation and progression but also establishes a solid foundation for designing targeted interventions aimed at specific spatial niches, disrupting critical cell–cell interactions, or remodeling the local microenvironment [12, 31, 32]. Furthermore, spatial multi‐omics integration is essential for bioinformatics analysis to elucidate mechanisms of therapeutic resistance, as they allow tracking of the spatial evolutionary trajectories of tumor clones under treatment pressure and their interplay with microenvironmental remodeling—such as altered immune cell composition and stromal reorganization—thereby providing novel strategies to overcome resistance [33].

### 2.2 Integrating MALDI‐Based Spatial Metabolomic, Lipidomic, and Partial Spatial Proteomic Data

Within the framework of spatial multi‐omics integration, the information provided by different omics platforms can be complementary, yet in certain contexts may also display “paradoxical” phenomena. Matrix‐assisted laser desorption/ionization (MALDI) mass spectrometry imaging serves as a typical example. MALDI imaging has distinct advantages in spatial metabolomics and spatial lipidomics, relying on laser irradiation of matrix‐coated tissue sections to desorb and ionize analytes. The resulting ions are detected by their mass‐to‐charge ratios (m/z), thereby enabling high‐throughput, label‐free visualization of the in situ spatial distribution of molecules within tissue slices [34]. This technology exhibits exceptional sensitivity and specificity for the abundant metabolites (e.g., amino acids, sugars, nucleotides, and energy metabolism intermediates) and lipid species (e.g., phospholipids, sphingolipids, and glycerolipids) present in the tumor microenvironment [35].

For example, in breast cancer research, MALDI imaging has clearly delineated the unique metabolite and lipid signatures of distinct functional tumor regions, such as proliferative cores, hypoxic necrotic zones, and invasive fronts. In proliferative areas, high levels of phosphorylated glycolytic intermediates (e.g., phosphoenolpyruvate) and phosphatidylcholine (PC) lipids were detected, reflecting active energy metabolism and membrane biogenesis demands [36]. By contrast, hypoxic necrotic regions exhibited accumulation of carnitine species and gradient distributions of specific sphingolipids (e.g., sphingosine‐1‐phosphate), indicating lipid metabolic reprogramming and potential signaling roles [35, 37]. These spatially resolved metabolite and lipid maps provide direct evidence for understanding energy supply, signal transduction, oxidative stress responses, and membrane dynamics within the tumor microenvironment. As such, they serve as an essential complement to spatial proteomics and spatial transcriptomics, since they directly capture the functional and biochemical states of cells.

However, attempts to apply MALDI mass spectrometry imaging (MSI) directly to spatial proteomics (i.e., intact protein detection) have encountered striking “paradoxical” phenomena and technical bottlenecks. This is primarily due to the intrinsic characteristics of proteins—large molecular weights (typically >10 kDa), complex higher‐order structures and post‐translational modifications, susceptibility to suppression during matrix crystallization, and much lower ionization efficiency compared with small molecules [38]. Although some studies have sought to address these challenges by optimizing matrix selection (e.g., employing ultrafine gold or silver nanoparticles), generating peptides in situ through enzymatic digestion (akin to MSI histology), or employing antibody‐assisted derivatization strategies (e.g., metal‐tagged antibodies) [39], such approaches either drastically limit the number of proteins detectable (low throughput) or compromise spatial resolution and specificity, rendering them less competitive than antibody‐based, highly multiplexed spatial proteomics technologies such as IMC, CODEX, or MIBI [40]. Consequently, when incorporated into spatial multi‐omics cancer studies, MALDI MSI contributes primarily to metabolomic and lipidomic dimensions, whereas spatial proteomic information typically relies on complementary antibody‐based imaging platforms. This “paradox” at the technical level underscores the necessity of integrating diverse spatial omics modalities and highlights their complementary value in unraveling tumor biology.

**FIGURE 5:**
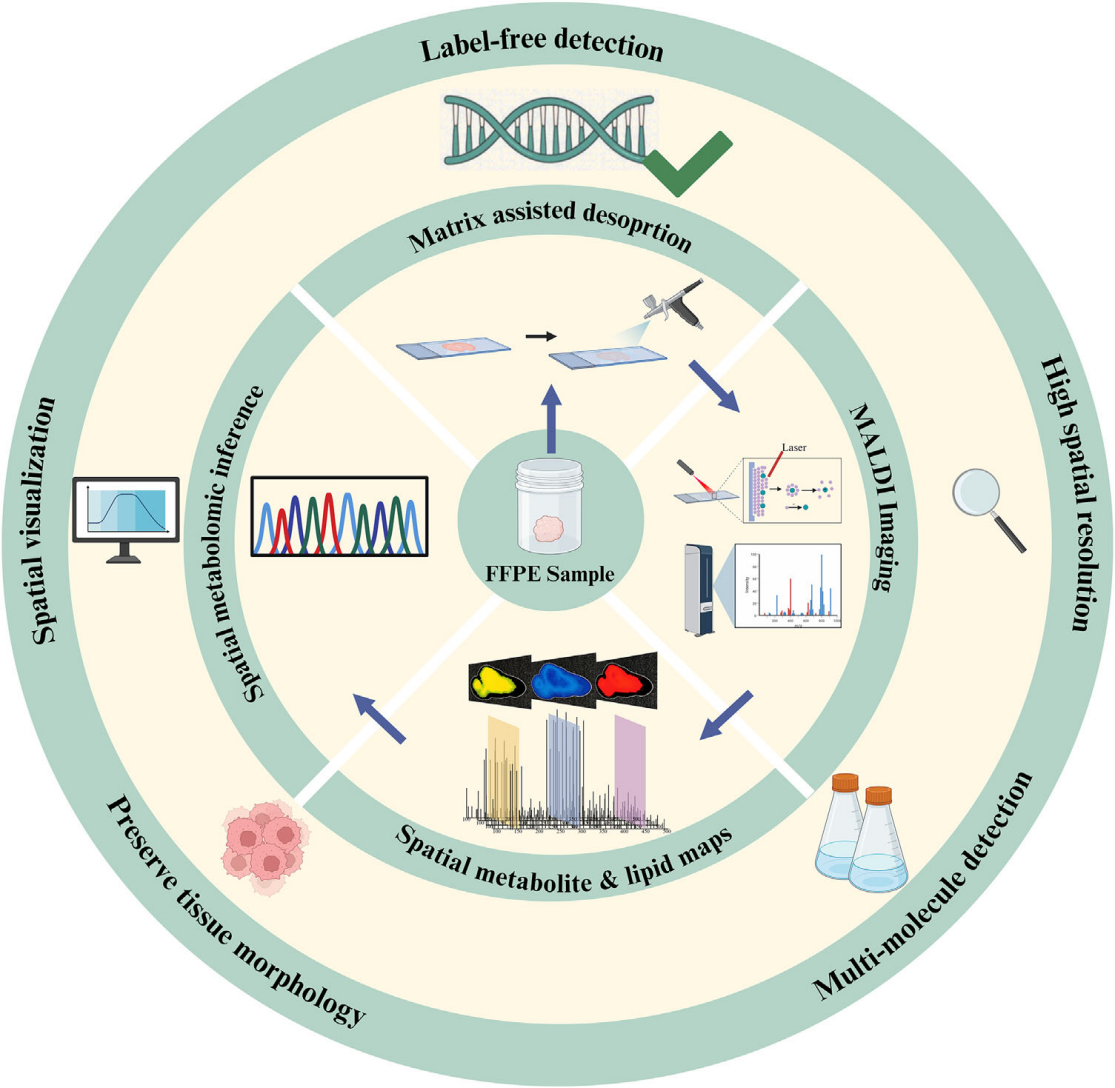
| Procedures and characteristics related to MALDI‐MS (matrix‐assisted desorption mass spectrometry) imaging based on FFPE samples. MALDI imaging technology enables MALDI‐MS to achieve high spatial resolution for tissue analysis. The label‐free nature of this approach allows simultaneous detection of multiple molecular species in a single measurement. This approach allows spatial mapping of metabolites and lipids, supports spatial metabolomic inference, and complements antibody‐based spatial proteomics platforms by providing metabolic and lipidomic dimensions within the tumor microenvironment.

The spatial distribution of metabolites and lipids (captured by MALDI and related imaging methods) is functionally and intimately connected to protein expression. For example, in renal cell carcinoma, MALDI imaging revealed abnormal regional accumulation of fumarate (driven by inactivating FH mutations), and this spatial metabolic signature was directly linked to local stabilization and activation of HIF‐1α protein (validated by immunohistochemistry or IMC), thereby driving angiogenesis and invasive phenotypes [41]. Protein data alone would be insufficient to infer such metabolic activity, whereas metabolomic data provided direct evidence. In prostate cancer, MALDI lipid imaging demonstrated enrichment of lysophosphatidic acid (LPA) at tumor margins. This spatial pattern was closely associated with high expression of the LPA receptor (LPAR1) detected in adjacent regions by spatial proteomics (e.g., CODEX), along with phosphorylation of downstream Rho/ROCK signaling proteins, together implicating the LPA–LPAR1 axis in promoting tumor cell migration and invasion [42]. Here, lipidomics delivered spatial information about ligands, while proteomics revealed receptor and effector states. In glioblastoma, spatial proteomics (e.g., MIBI) identified abnormally elevated expression and phosphorylation of the c‐Met receptor tyrosine kinase in defined tumor regions. Integration with MALDI metabolomic data further revealed significant alterations in arginine metabolites (e.g., agmatine) in the same regions, suggesting that c‐Met signaling may influence tumor immune evasion through regulation of arginine metabolism [43]. Protein expression thus indicates potential regulators, whereas metabolite alterations signify their functional consequences. The procedural framework and defining characteristics of MALDI‐MS imaging are summarized in Figure 5. As depicted, MALDI imaging operates through label‐free detection principles, enabling simultaneous identification of multiple molecular species in a single measurement. This approach facilitates spatial mapping of metabolites and lipids, supports spatial metabolomic inference, and serves as a complementary modality to antibody‐based spatial proteomics platforms by capturing the metabolic and lipidomic dimensions of the tumor microenvironment.

Taken together, despite its limitations in direct intact protein detection, MALDI's powerful capabilities in spatial metabolomics and lipidomics make it an indispensable complement to high‐dimensional spatial proteomics and transcriptomics. Multilayered integrative analyses can therefore more comprehensively resolve molecular networks and spatial regulatory mechanisms within the tumor microenvironment—from gene expression to protein function to metabolic activity—exposing paradoxical associations invisible to single‐omics approaches (e.g., regions of high protein expression lacking corresponding metabolic substrates). This provides a more systematic spatial molecular atlas for understanding tumor heterogeneity, therapeutic resistance, and the design of precision interventions. Looking ahead, the development of computational biology methods and multimodal imaging platforms that more effectively integrate MALDI‐derived metabolomic/lipidomic data with other spatial omics datasets (particularly high‐dimensional spatial proteomics) will be crucial for overcoming current “paradoxes” and maximizing the complementary value of multi‐omics integration [44].

## 3 Deep Visual Proteomics (DVP): A Milestone Advancement in Spatial Proteomics

**FIGURE 6:**
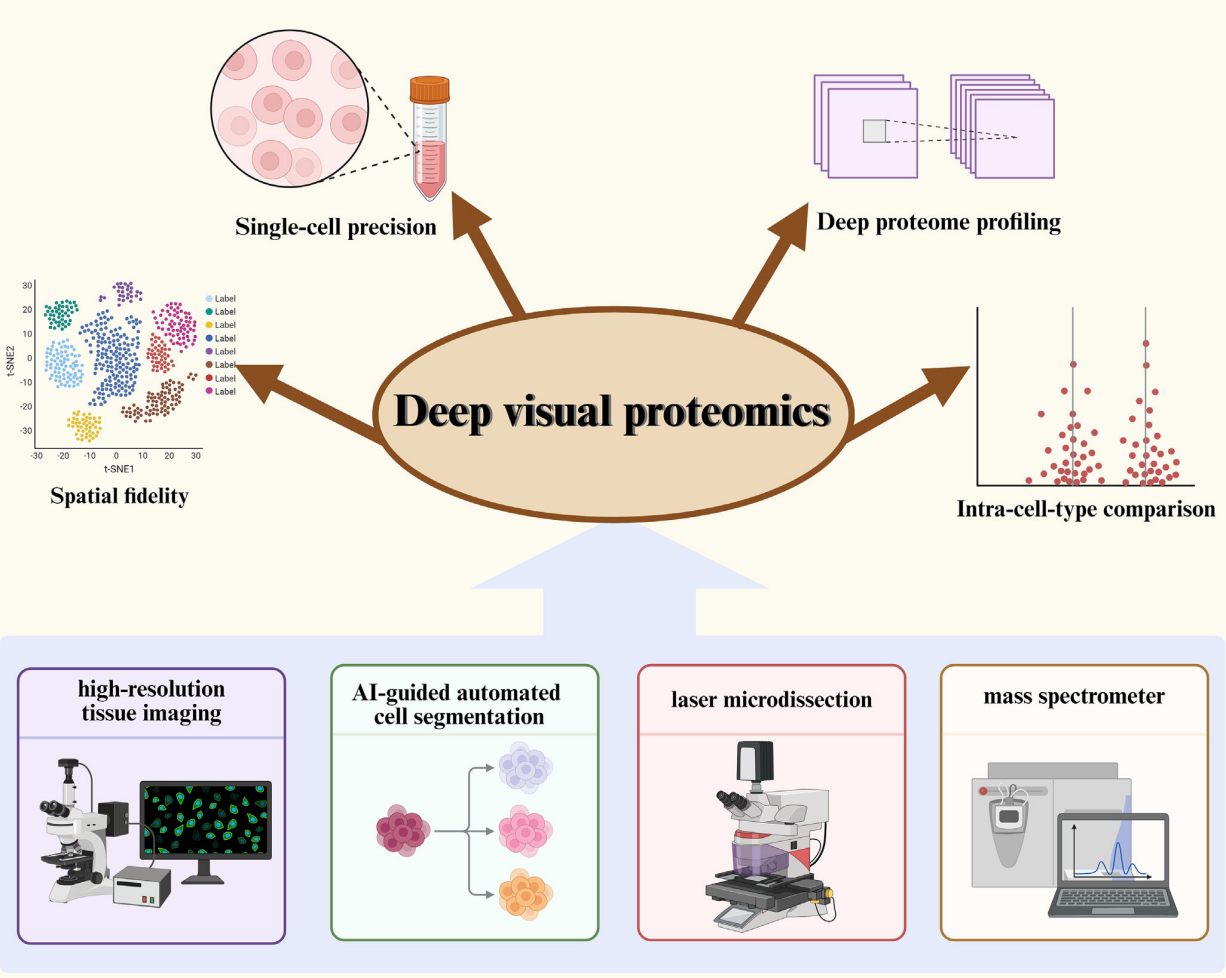
Principle and key advantages of Deep Visual Proteomics (DVP). DVP integrates high‐resolution tissue imaging, AI‐guided cell segmentation, laser microdissection, and ultrasensitive mass spectrometry to achieve phenotype‐specific and spatially resolved proteomic profiling. This approach preserves spatial tissue context while providing deep, unbiased, and highly sensitive protein quantification at the single‐cell or cell‐type level.

Over the past decade, spatial proteomics technologies have undergone revolutionary advancements—from early immunohistochemistry and in situ hybridization, to mass spectrometry‐based spatially resolved approaches, and now to deep visual proteomics (DVP). Every technological iteration has substantially expanded our understanding of protein distribution patterns within the tissue microenvironment (see “MS‐Based Spatial Proteomics”‐ “DVP” in Table S1). The advent of DVP marks a new era for spatial proteomics: it transcends simple protein localization or limited target detection, enabling deep, unbiased proteomic profiling for specific cell populations while preserving tissue integrity and spatial context. DVP integrates mass spectrometry with high‐resolution tissue imaging, AI‐guided automated cell segmentation, and laser microdissection, followed by ultrasensitive proteomic analysis of phenotypically defined cells [45, 46, 47]. This innovation dramatically enhances the precision of spatial proteomics and allows direct comparison of proteomic signatures within the same cell type, all while retaining both spatial context and molecular features [47]. The operational principle and core advantages of DVP are schematized in Figure 6. The workflow integrates four key components: (i) high‐resolution tissue imaging for morphological assessment, (ii) AI‐guided cell segmentation for precise phenotypic classification, (iii) laser microdissection for targeted cell isolation, and (iv) ultrasensitive mass spectrometry for deep proteomic profiling. This multi‐step approach preserves spatial tissue context while achieving unprecedented depth in protein quantification—approximately 1700 proteins per cell—at the single‐cell or cell‐type level.

The application of DVP in melanoma research has vividly demonstrated its powerful capacity to elucidate the molecular mechanisms of tumor progression. Using DVP, researchers analyzed archived melanoma tissue specimens from different pathological stages—including benign nevi, melanoma in situ, invasive melanoma, and metastatic lesions—to construct a dynamic proteomic atlas of melanoma progression [47]. This approach revealed the proteomic transitions underlying the progression of normal melanocytes into invasive malignant tumors and precisely mapped spatial alterations in key signaling pathways and molecular networks associated with cancer progression^3^ ^5^. Building upon this framework, single‐cell deep visual proteomics (scDVP) has further extended these methodologies to the analysis of complex and/or poorly characterized proteomes, achieving unprecedented depth with the quantification of ∼1700 proteins per cell.

In liver research, scDVP has revealed protein expression gradients that not only validate classical models of lobular metabolic zonation (e.g., the differential distribution of glycogen synthesis and detoxification functions across distinct lobular regions), but also unexpectedly uncovered aberrant spatial patterns closely associated with the pathological progression of chronic liver diseases [48]. In a non‐alcoholic fatty liver disease (NAFLD) model, scDVP quantitatively delineated proteomic remodeling in zone 3 of the hepatic lobule (peri‐central vein), characterized by the accumulation of lipid metabolism–related enzymes (such as fatty acid–binding protein FABP1 and lipid droplet–associated protein PLIN2) and endoplasmic reticulum stress markers (such as GRP78/BiP), alongside a marked downregulation of antioxidant enzymes (e.g., glutathione peroxidase GPX1) [49]. This spatially resolved imbalance in protein expression provides direct molecular evidence of oxidative stress and lipid peroxidation damage occurring in the disease's core regions, offering new avenues for spatially targeted interventions against metabolic dysregulation.

Meanwhile, with the increasing maturity of automated laser microdissection platforms coupled with high‐sensitivity mass spectrometers, scDVP is being progressively applied to the large‐scale construction of spatial proteomic atlases from clinical pathology samples [50]. For example, in a retrospective study using breast cancer tissue microarrays (TMAs), researchers employed scDVP to systematically analyze thousands of single cells from tumor core regions, invasive fronts, and adjacent normal ductal areas across different molecular subtypes (e.g., Luminal A, HER2^+^, and triple‐negative breast cancer). This study not only successfully mapped the quantitative spatial distribution networks of key signaling proteins driving malignant phenotypes (such as ERα, HER2, EGFR, and Ki‐67) within the tumor microenvironment, but also integrated these spatial proteomic features using machine learning algorithms to build a prognostic prediction model that surpassed traditional histological subtyping in accuracy and clearly outperformed models based solely on morphology or bulk proteomic data [51]. These findings highlight spatially resolved single‐cell proteomics as an emerging bridge between fundamental tumor biology and precision theranostics. They also offer strong potential for clinical translation, for applications such as deciphering tumor heterogeneity, identifying microenvironmental drivers, and developing individualized therapeutic strategies. In the future, the integration of scDVP with spatial metabolomics and multiplexed fluorescence imaging, alongside the development of more powerful algorithms for integrating spatial multi‐omics data, holds great promise for reconstructing the spatiotemporal dynamics of tumor initiation, evolution, and therapeutic response at single‐cell resolution [52].

Spatial proteogenomics uniquely enables the investigation of fundamental biological questions in cancer research. These include: (i) phenotypic and functional heterogeneity within the tumor microenvironment, where spatially resolved protein expression reveals immune exclusion, exhaustion, or activation states [53]; (ii) mechanisms of therapeutic resistance, by tracking spatially localized signaling rewiring and clonal evolution under treatment pressure [54]; (iii) identification of actionable targets and neoantigens, particularly from the dark proteome, within defined spatial niches [55]; and (iv) tumor initiation and progression, where spatial gradients of protein activity and metabolic adaptation provide mechanistic insights beyond genomic alterations alone [56, 57]. By explicitly linking spatial molecular patterns to biological function, spatial proteogenomics transforms descriptive maps into actionable cancer biology.

## 4 Spatial Proteomic Strategies for Dark Proteome Detection

### 4.1 Definition and Key Challenges of the Dark Proteome

“Dark proteome” consists of proteins that are theoretically encoded in the genome‐transcriptome but whose sequence and structural information remain invisible to traditional detection approaches [58]. These proteins fall into several categories: (i) unstable or rapidly degraded molecules with very short half‐lives [59]; (ii) proteins expressed at extremely low abundance, below the detection limits of standard mass spectrometry [60, 61]; and (iii) noncanonical proteins arising from alternative mechanisms such as noncanonical open reading frames (uORFs) [62, 63], alternative translation initiation sites, or non‐standard splicing events. Additionally, (iv) proteins that serve as substrates of regulatory pathways — particularly those subject to nonsense‐mediated mRNA decay (NMD) [62] — are also considered part of the dark proteome. Collectively, these characteristics define the dark proteome as a domain of biology that is technically challenging but also rewarding to study.

The spatial characterization of the dark proteome faces three major challenges. First, dark proteins are often present only at trace levels in the sample and/or restricted to individual cells. Conventional mass spectrometry methods encounter substantial challenges when detecting trace proteins, particularly those present only in individual cells: because extraction, digestion, and cleanup are performed separately and involve repeated liquid handling, evaporation, and adsorption to container surfaces, a large fraction of the original sample is lost before it reaches the chromatography and ionization stages. For low‐abundance proteins, this loss often reduces their concentration below the detection threshold or eliminates them completely [64]. Second, dark proteins frequently contain unannotated, degradation‐prone regions or post‐translational modifications (PTMs). Third, antibodies corresponding to many dark proteins have not yet been discovered or designed.

#### 4.1.1 Feasible Technical Pathways to Address the Above Challenges

**FIGURE 7:**
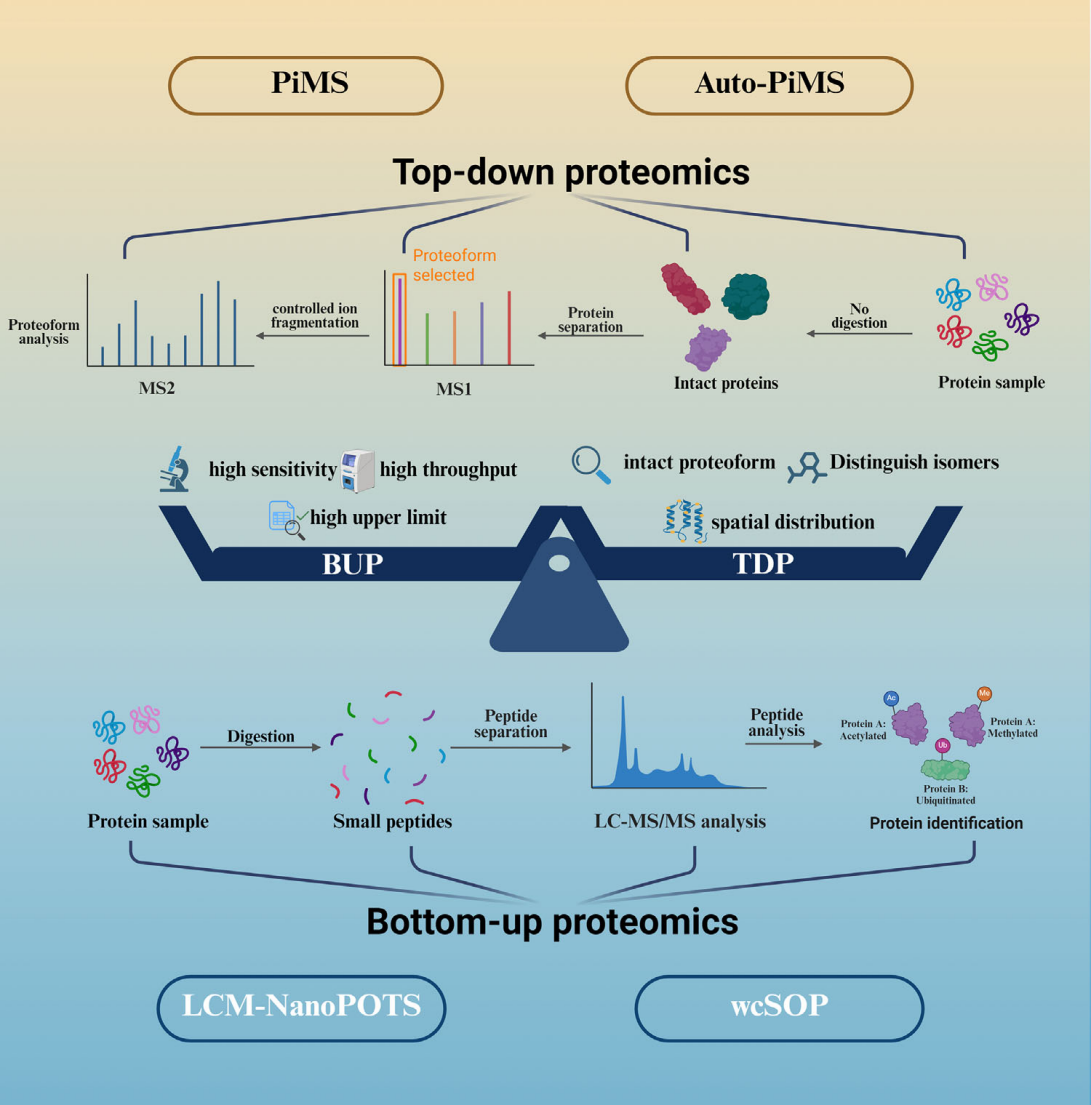
| Schematic comparison of bottom‐up and top‐down proteomics strategies. The “dark proteome” comprises proteins that are theoretically encoded in the genome–transcriptome but remain undetectable by traditional proteomics workflows due to instability, low abundance, or noncanonical translation mechanisms. To overcome these challenges, two complementary antibody‐free strategies have been developed: bottom‐up proteomics (BUP) and top‐down proteomics (TDP). In BUP, proteins are enzymatically digested into peptides prior to LC–MS/MS analysis, offering high sensitivity, high throughput, and broad dynamic range, as demonstrated by NanoPOTS and LCM–NanoPOTS platforms. In contrast, TDP introduces intact proteins directly into the mass spectrometer, enabling the identification of proteoforms and isomeric species while retaining spatial distribution information, as exemplified by Proteoform Imaging Mass Spectrometry (PiMS) and its automated version Auto‐PiMS. Despite its lower throughput, TDP provides unparalleled capability in resolving full information of proteoforms (structure and modifications). Together, these complementary approaches provide a powerful framework for uncovering spatial distributions and structural diversity within the dark proteome.

Considering the above challenges associated with spatial characterization of the dark proteome, antibody‐based omics approaches remain limited, as they rely on specific antibodies and often fail to capture the full information of proteoforms (a protein molecule with a defined sequence, structure, and post‐translational modifications) [65]. To overcome these challenges in dark proteome analysis, two mass spectrometry‐based, antibody‐independent strategies have emerged: bottom‐up proteomics (BUP) [66] and top‐down proteomics (TDP) [67] (Figure 7 and “MS‐Based Spatial Proteomics” in Table S1).

**BUP** refers to a workflow in which proteins are digested by proteolytic enzymes (e.g., trypsin) prior to liquid chromatography–mass spectrometry (LC–MS) analysis. The resulting peptides are ionized, fragmented, and analyzed, and protein sequences are reconstructed by database searching and peptide assembly [68]. This “small‐to‐large” reconstruction principle underlies the name “bottom‐up.” Advantages of BUP include: (i) reliance on well‐established peptide databases and search engines, which have been widely adopted even prior to the development of TDP; (ii) substantially higher throughput than TDP, making it suitable for large‐scale proteomic studies; and (iii) fewer isotopic atoms, resulting in simpler spectra, which leads to higher detection sensitivity and a higher upper limit for the molecular weight of detectable proteins [67]. Nevertheless, BUP requires enzymatic digestion during sample preparation [67, 69]. As a result, it shows inherent limitations in analyzing highly hydrophobic proteins such as membrane proteins, structurally unstable or degradation‐prone proteins, and proteins carrying labile modifications (e.g., phosphorylation, glycosylation, sulfation, or ADP‐ribosylation). For these classes, BUP is often insufficient to fully reconstruct intact proteoforms or to resolve their complete structural information [67, 69].

**TDP**, by contrast, bypasses proteolysis and introduces intact proteins or protein complexes directly into the mass spectrometer. Primary mass spectra provide molecular weight and charge‐state information, while subsequent MS stages with controlled fragmentation (e.g., ETD, ECD, UVPD) enable structural characterization. Same as BUP, database searching and fragment assembly are also required by TDP in order to obtain full proteoform information [67]. TDP is capable of directly resolving the overall features of proteoforms, particularly those with labile post‐translational modifications that may be lost during BUP [67]. However, TDP suffers from lower throughput compared with BUP and remains constrained by the requirements for ultrahigh‐resolution instrumentation and computationally intensive spectral interpretation [67, 68].

### 4.2 Applying Bottom‐Up Proteomics (BUP) to Spatial Analysis of the Dark Proteome

This section primarily introduces two spatial applications of bottom‐up proteomics (BUP) platforms: NanoPOTS [64] and its spatial implementation, LCM‐NanoPOTS [70, 71, 72], as well as wcSOP [73]. All these approaches are capable of detecting >4000 proteins at the scale of ∼100 cells, thereby providing high‐throughput capacity for identifying structurally stable dark proteins.

#### 4.2.1 Single‐Cell Mass Spectrometry Enabled by the NanoPOTS Nanoscale Sample‐Processing Platform and Spatial Proteomics Supported by LCM–NanoPOTS

One solution for single‐cell proteome quantification is the live single‐cell mass spectrometry (sc‐MS) powered by NanoPOTS (Nanodroplet Processing in One Pot for Trace Samples) platform, developed by Ying Zhu et al. from Ryan T Kelly research group [64]. NanoPOTS platform comprises a glass chip with nanowell array, a reagent cocktail, a robotic microfluidic dispensing system capable of handling picoliter volumes [74], and silica capillaries for sample collection. By integrating all the required sample preparation steps in a “micro pot” droplet inside a nanowell, the necessary starting volume is reduced from about 0.5 milliliters to ∼200 nanoliters, typically with a few or tens of cells inside. After NanoPOTS processing, Kelly and colleagues used ultra‐low flow nanoLC columns with an inner diameter of approximately 30 micrometres; the combination of very narrow diameter and very low flow rate improves peptide separation and electrospray ionization efficiency, enabling more low‐abundance peptides to be detected by the mass spectrometer. Thirdly, in earlier work [75], Kelly's group developed a droplet‐based nano‐electrospray ionization method in which extremely small aqueous droplets are generated within an immiscible oil phase, which protects them until delivery; at the emitter only the aqueous phase enters the electrospray, thus avoiding dilution by the bulk flow, maximizing ion utilization efficiency, and enabling stable detection even for ultra‐small sample amounts. Leveraging these innovations together with the MaxQuant algorithm [76], NanoPOTS achieves a remarkable capability to identify approximately 3000 proteins from as few as ∼10 cells without the use of antibodies or labels, and further enables proteome profiling at the level of individual cells [64].

To provide NanoPOTS with spatial resolution and the ability to uncover tissue heterogeneity, Ying Zhu and colleagues from the Ryan T. Kelly group developed the LCM‐NanoPOTS automated platform by integrating laser capture microdissection (LCM) with NanoPOTS [70]. In this approach, 200 nL droplets of DMSO buffer (capable of dissolving cellular lipids) were preloaded into each nanowell of the NanoPOTS chip, after which the chip was mounted on a standard microscope slide adapter (SlideCollector 48, Carl Zeiss MicroImaging) and positioned on the robotic arm of the LCM system. Finally, tissue sections excised by laser pressure were catapulted into the droplets. After tissue is laser‐dissected and ejected into droplets, digestion enzymes and other reagents can be introduced using a capillary; alternatively, the digestion step can be omitted to retain intact proteins for TDP analysis [69]. This platform achieved an 87% success rate of accurate deposition at a minimum cutting diameter of 20 µm and enabled the identification of approximately 100 proteins from mouse tissue pieces with a diameter of 100 µm and a thickness of 12 µm, corresponding to 10–18 cells. Subsequently, the Ryan T. Kelly group implemented two rounds of optimization of the LCM‐NanoPOTS platform in 2020 [72] and 2023 [71]. Following the first optimization, more than 2000 proteins were identified from mouse tissue voxels of the same size (100 µm in diameter and 12 µm in thickness) [72], while the second optimization enabled the detection of over 6000 proteins from human islet tissue sections with a thickness of 10 µm and a surface area of 500 000 µm^2^ [71].

#### 4.2.2 Wet Collection of Single Microscopic Tissue Voxels and Surfactant‐Assisted One‐Pot Processing (wcSOP)

Reta B. Kitata and colleagues from Tujun Shi's group invented a one‐pot‐based method termed wet collection of single microscale tissue voxels and Surfactant‐assisted One‐Pot voxel processing (wcSOP) [73]. Similar to LCM‐NanoPOTS, this method employs LCM to cut tissue (hereinafter referred to as “voxels”) and eject them into a droplet, where protein digestion and other processing steps are carried out. The main differences between wcSOP and LCM‐NanoPOTS are as follows: (i) wcSOP uses a PCR tube cap with a diameter of 5.4 mm containing a 25 µL droplet, ensuring that tissue voxels ejected by LCM are fully immersed and digested; in contrast, LCM‐NanoPOTS employs a nanowell with a diameter of 1.2 mm on a NanoPOTS glass chip containing a 200 nL droplet. Thus, the larger surface area of the PCR tube cap in wcSOP reduces the precision requirement of LCM compared with NanoPOTS; (ii) each PCR tube cap accommodates only one droplet, whereas a NanoPOTS chip can contain multiple droplets; (iii) after digestion, droplets in wcSOP can be transferred from the tube cap to a vial by centrifugation for storage, enabling repeated and multiple analyses of the proteome; (iv) wcSOP does not require expensive custom glass chips or specialized microfluidic equipment, thereby reducing training and equipment costs. However, the original manuscript [73] did not demonstrate how multiple tissue blocks could be sequentially ejected and collected into multiple PCR tube caps. Therefore, prior to automation of the LCM‐PCR tube cap collection step, the analytical throughput and speed of wcSOP are still inferior to those of automated LCM‐NanoPOTS. Moreover, the LCM‐NanoPOTS platform allows TDP analysis by bypassing enzymatic digestion [69], whereas wcSOP does not provide this option.

### 4.3 Top‐Down Proteomics (TDP) Represented by Proteoform Imaging Mass Spectrometry (PiMS)

In this section, we introduce one of the most widely used spatial applications of top‐down proteomics (TDP)—Proteoform Imaging Mass Spectrometry (PiMS) [65] and its automated version, Auto‐PiMS [77]. In brief, PiMS is a combination of nanospray desorption electrospray ionization (nano‐DESI) [78] and individual ion mass spectrometry (I^2^MS) [79]. The core steps of PiMS involve using a dual‐capillary nano‐DESI solvent bridge to scan across tissue sections in a point‐by‐point fashion, dissolving protein molecules, followed by I^2^MS analysis [79]. Specifically, two capillaries form a stable nanoscale solvent micro‐bridge; when this bridge contacts the tissue surface, the solvent selectively desorbs and solubilizes proteins and other biomolecules from the local microenvironment [78]. The solubilized proteins are then transported into the electrospray capillary, where they acquire multiple charges under nano‐ESI conditions, and are subsequently captured and resolved as individual ions with high‐resolution Orbitrap mass spectrometry [79].

Unlike conventional BUP strategies, PiMS allows direct detection of intact proteins without chromatographic separation or enzymatic digestion, and, when combined with fragmentation approaches such as electron capture dissociation (ECD), electron transfer dissociation (ETD), or ultraviolet photodissociation (UVPD), it provides comprehensive characterization of proteoforms, including their intact masses, sequence fragments, and post‐translational modifications. By sequentially scanning the tissue surface, PiMS reconstructs spatial proteoform distributions, thereby establishing a genuine spatial proteomics platform. This approach is particularly advantageous for probing the dark proteome, as many low‐abundance, hydrophobic, or heavily modified proteins that are typically inaccessible in bottom‐up workflows can be captured and characterized at the proteoform level by PiMS [65]. A common challenge of TDP is the limited upper mass range for detection; however, PiMS effectively extends this upper mass limit while maintaining relatively high analytical throughput [65].

Building upon the foundation of PiMS, Auto‐PiMS [77] represents a significant advancement by incorporating automated control of the solvent bridge, programmed scanning trajectories, and integrated data acquisition workflows [77]. This automation not only enhances throughput and reproducibility in large‐area tissue imaging but also minimizes variability and positional errors introduced by manual operation. Furthermore, Auto‐PiMS leverages optimized mass spectrometric acquisition schemes and data processing pipelines to accelerate the generation of high‐dimensional proteoform imaging data while preserving the resolution and precision of individual ion detection. Consequently, Auto‐PiMS provides a more robust and standardized platform for high‐throughput spatial proteomics.

### 4.4 FLASHDeconv: An Optimized Algorithm for Simplifying TDP–MS Spectral Deconvolution

In spatial proteomics, the elucidation of proteoform‐level information relies primarily on mass spectrometry rather than image‐based approaches such as multiplexed immunofluorescence (mIF) or multiplexed immunohistochemistry (mIHC). Two major strategies exist: top‐down proteomics (TDP‐MS), which directly analyzes intact proteins, and bottom‐up proteomics (BUP‐MS), which reconstructs full‐length sequences from peptide fragments in de novo or otherwise unidentified proteins. Among these, TDP‐MS is particularly critical, as it requires measurement of intact protein mass‐to‐charge (m/z) ratios at the MS1 level. The presence of numerous stable isotopes in proteins results in highly convoluted spectral patterns not only at MS1 but also at subsequent MS2 and MS3 stages. This intrinsic complexity substantially increases the difficulty of proteoform identification when using conventional TDP software packages such as ProSightPC [80, 81, 82, 83] or MSPathFinder [84, 85], thereby constituting a major obstacle for the application of TDP to spatial proteomics.

FLASHDeconv [86] addresses this bottleneck by implementing a highly efficient three‐step deconvolution framework [87]. The first step, decharging, determines the charge states—or equivalently, the masses—of peaks. The principal speedup of FLASHDeconv is achieved here through a simple yet powerful transformation: peak positions (m/z) are mapped into log(m/z) space, where mass‐dependent charge patterns are converted into a universal, mass‐independent pattern. Identifying occurrences of this universal pattern becomes computationally trivial, requiring only a single convolution calculation, while harmonic artifacts are efficiently reduced by minor modifications of the same approach. The second step, deisotoping, involves finding theoretical isotope patterns predicted by the averagine model around charge‐determined peaks. Corresponding isotope peaks are collected, projected into mass space, and centroided to determine the monoisotopic mass. This step alleviates complications arising from isotopically unresolved signal regions and aggregates intensities from overlapping peaks. Finally, the feature finding step traces deconvoluted masses along retention time (RT) using a robust mass trace detection algorithm (Kenar et al., 2014), thus defining complete mass features for downstream analysis. The complementary strategies of bottom‐up and top‐down proteomics for dark proteome spatial analysis are schematically compared in Figure 7. As illustrated, bottom‐up proteomics (BUP) achieves high sensitivity and throughput through enzymatic digestion and peptide‐level analysis, as exemplified by NanoPOTS and LCM‐NanoPOTS platforms. In contrast, top‐down proteomics (TDP), represented by PiMS and Auto‐PiMS, bypasses digestion to analyze intact proteins, preserving proteoform‐level information including post‐translational modifications and enabling spatial distribution mapping. The selection between these approaches depends on whether comprehensive protein coverage (BUP) or detailed proteoform characterization (TDP) is prioritized.

By integrating these steps, FLASHDeconv substantially reduces the computational burden of TDP deconvolution while maintaining high accuracy, making it well‐suited for large‐scale spatial proteomics datasets. Its importance lies in enabling more tractable analysis of TDP spectra and providing reliable inputs for proteoform identification and quantification [88]. Nonetheless, several caveats remain. FLASHDeconv is not a deep learning–based general model but rather an optimization‐driven algorithm, and it may still struggle under conditions of extremely low abundance or high background noise. Furthermore, deconvolution represents only the first step in protein characterization and must be complemented by database searching or sequence matching for definitive identification. Its utility is thus most pronounced in intact protein analysis, with limited impact on bottom‐up peptide‐centric workflows where isotopic complexity is far less severe.

## 5 Integration of Spatial Proteomics and Artificial Intelligence: From Technical Challenges to Intelligent Model Applications

### 5.1 The Macroscopic Significance of Integrating Spatial Proteomics With AI

The rapid advancement of spatial proteomics has been fueled by continuous technological innovation and the deep integration of multidisciplinary approaches. Within this progression, artificial intelligence (AI) has gradually emerged as one of the key driving forces propelling the transition of the field from basic research to clinical application. The integration of AI with spatial proteomics (“Traditional ML‐ and AI‐based Proteogenomics Methods” in Table S1) not only facilitates the discovery of predictive biomarkers but also enables more accurate clinical stratification, thereby significantly advancing the development of personalized cancer therapy [89, 90]. This deep convergence enhances the interpretability of multimodal data and provides unprecedented scientific insights and translational potential for precision oncology.

### 5.2 Technical Challenges in Spatial Proteomics and the Value of AI Intervention

Despite these advances, current spatial proteomics technologies remain constrained by limitations in resolution and sensitivity, particularly with respect to integrative analyses at the proteome level, which hinder comprehensive characterization of intratumoral protein expression heterogeneity. Traditionally, morphology‐based approaches have been used to explore biological variation within tumors and to define relatively homogeneous ecological niches for deeper molecular analysis. However, these approaches rely heavily on manual annotation by pathologists, leading to subjectivity and qualitative bias. Against this backdrop, artificial intelligence—especially advances in unsupervised learning—offers new avenues for automated and objective tumor region segmentation, enabling high‐throughput identification of tumor subpopulations and deepening our understanding of tumor biology and therapeutic resistance [91].

Furthermore, spatial proteomics provides region‐specific protein expression data that serve as a high‐quality foundation for AI model training. In turn, AI—through advanced tools such as unsupervised learning and regression modeling—overcomes the limitations of manual analysis by enabling quantitative modeling of intratumoral heterogeneity (e.g., tri‐axial models) and precise prediction of clinically relevant features such as drug resistance and patient survival [92]. This synergy not only deepens our understanding of tumor biology but also provides robust data and methodological support for designing personalized combination therapies targeting tumor heterogeneity. For example, studies employing multi‐omics integration tools such as *iCluster* have identified three molecular subtypes of esophageal squamous cell carcinoma, integrating metabolic modeling and machine learning to predict radioresistance. In parallel, joint analyses of single‐cell and spatial transcriptomic data have revealed the spatial distribution and interaction patterns of distinct cell types within the tumor microenvironment [93].

In addition, AI technologies further facilitate the integration of multimodal data, encompassing genomic, transcriptomic, and spatial proteomic layers. By systematically mining these multidimensional datasets through advanced algorithms, researchers can uncover potential associations between protein expression patterns, tumor biology, and therapeutic resistance, thereby informing the design of combination therapies. For instance, imaging mass cytometry (IMC) has provided high‐resolution spatial and proteomic maps of the tumor microenvironment in intrahepatic cholangiocarcinoma (iCCA), while AI‐driven deep learning models have extracted prognostically significant spatial features from these complex datasets, achieving superior predictive performance [94]. This “multi‐omics–AI” integrative framework offers a novel perspective for molecular subtyping, prognostic evaluation, and therapeutic strategy selection in iCCA.

More specifically, spatial proteomics provides spatially resolved information on protein distribution within the tissue microenvironment, while AI transforms raw data into interpretable biological insights through image analysis, phenotype classification, and multi‐source data integration. For example, AI‐assisted deep visual proteomics (DVP), combined with ultrahigh‐sensitivity mass spectrometry techniques such as diaPASEF, enables quantification of thousands of proteins at the single‐cell level and even captures low‐abundance nuclear phenotypic features of rare cell types. This approach can be directly applied to archived clinical specimens—such as formalin‐fixed paraffin‐embedded (FFPE) tissues—allowing the elucidation of molecular mechanisms underlying disease progression while preserving spatial architecture, thus providing new avenues for target discovery and mechanistic analysis [47]. In one study, DVP was used to identify protein biomarkers associated with colorectal cancer recurrence and to establish a risk‐stratification framework based on molecular features, effectively overcoming the limitations of traditional methods that rely heavily on manual assessment and fail to link morphological phenotypes with molecular expression [95].

### 5.3 Multilevel Analytical Capabilities and Key Models of AI in Spatial Proteomics

**FIGURE 8 |:**
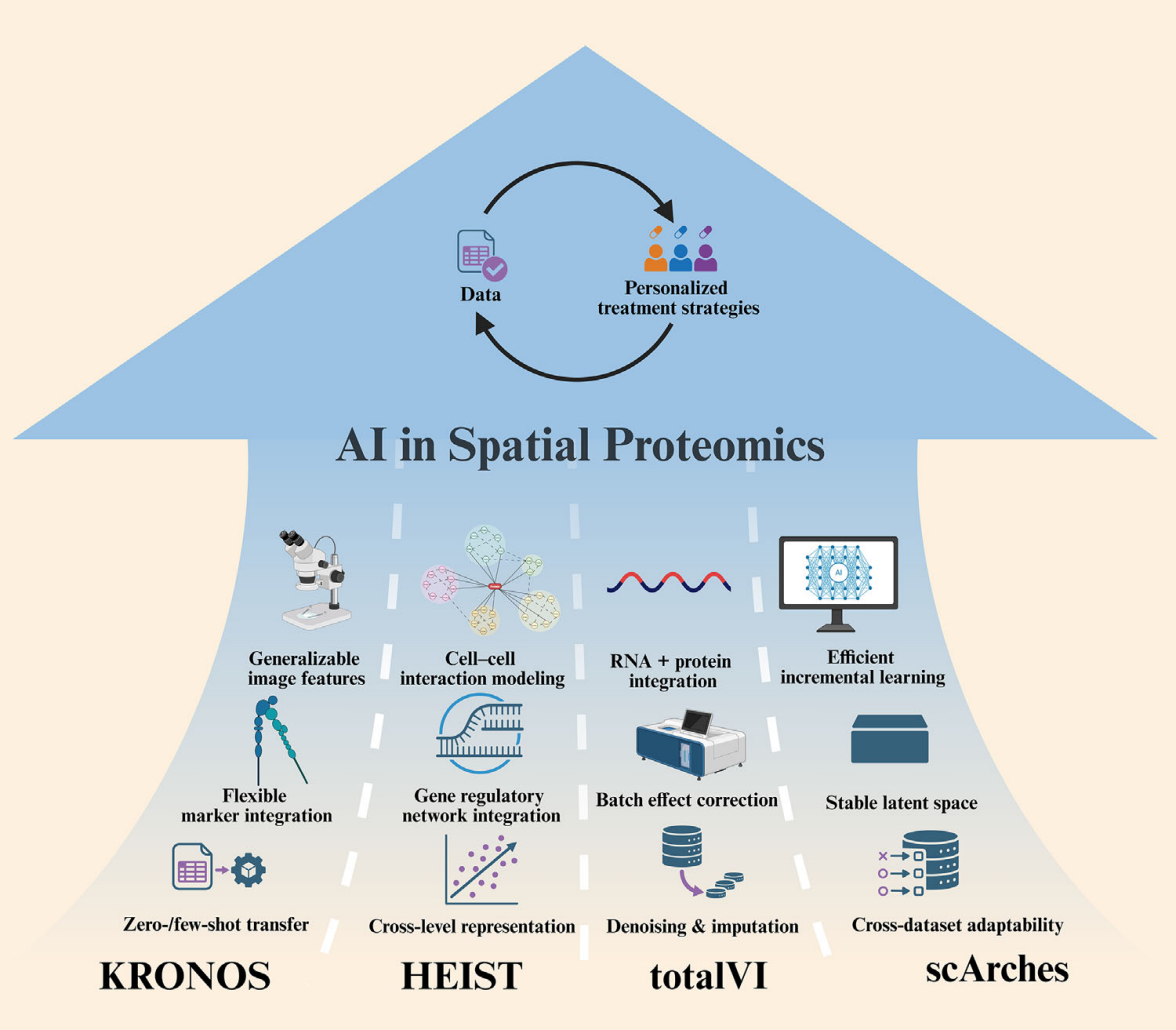
Artificial intelligence (AI) models driving advances in spatial proteomics. State‐of‐the‐art AI algorithms in spatial proteomics leverage data from multi‐channel stained images or pre‐processed spatial mass spectrometry datasets to enable multi‐scale biological interpretation—from single cells to tissue‐level architectures. Shown are four representative models and their key innovations. KRONOS, a vision transformer–based foundation model pretrained on multiplexed imaging data, extracts generalizable image features and supports flexible marker integration and zero‐/few‐shot transfer for cross‐marker analysis. HEIST, a hierarchical graph transformer, jointly models cell–cell interactions and gene regulatory networks (GRNs), achieving cross‐level representation that links molecular regulation to spatial context. totalVI, an autoencoder‐based framework, performs RNA–protein integration, batch effect correction, and denoising and imputation for multimodal datasets. scArches enables efficient incremental learning, stable latent representations, and cross‐dataset adaptability, facilitating large‐scale cohort integration. Together, these AI models advance the integration of spatial proteomics with spatial transcriptomics and histopathology, supporting data‐driven personalized treatment strategies.

Currently, mainstream artificial intelligence algorithms applied to spatial proteomics typically start from multi‐channel stained images or pre‐processed spatial mass spectrometry data. This is because the upstream steps—such as image acquisition, multichannel fluorescence intensity quantification, and proteoform identification from mass spectrometry data—are already supported by mature open‐source or commercial non‐deep‐learning software, including ZEISS ZEN and FlashDeconv [86]. State‐of‐the‐art (SOTA) models for spatial proteomics are now capable of addressing questions across multiple scales, ranging from protein markers to single cells, spatial spots, spatial patches, and even whole slide images (WSIs) [96]. These models enable not only microscopic‐to‐macroscopic biological insights, but also clinical diagnosis, synthetic data generation, denoising, and multi‐dataset integration [97]. More encouragingly, recent years have witnessed the emergence of multi‐purpose foundation models, opening new directions for spatial omics. In this section (Figure 8), we highlight the multi‐purpose foundation models KRONOS [98] and HEIST [99] for clinical diagnosis and data generation, as well as the autoencoder‐based model totalVI and its surgery‐based retraining strategy scArches, which facilitate the integration of spatial proteomics with spatial transcriptomics, H&E histology, and other multimodal datasets, as well as denoising and integration across diverse cohorts.

In the integration of spatial omics and pathology, histological images (such as H&E, mIHC, or mIF‐stained slides) have long served as the core source for interpreting tissue architecture and cellular distribution [100]. For decades, both the research and clinical communities have relied on manual annotations by expert pathologists or on traditional segmentation‐based spatial proteomics pipelines to analyze such images for scientific discovery and pathological diagnosis [101]. However, when manual annotation of histological images is not harmonized through systematic coordination under multi‐institutional consortia, combined with rigorous training and accreditation mechanisms, it often results in significant subjectivity and poor reproducibility, thereby limiting its utility in large‐scale studies and cross‐cohort comparisons [102]. More critically, even in standardized spatial proteomics pipelines, existing methods typically rely on cell segmentation followed by single‐marker thresholding or rule‐based gating mechanisms to assign cell phenotypes [103]. While effective in identifying canonical cell types, many approaches implicitly assume that the spatial distributions of individual markers are independent, thereby neglecting biologically meaningful spatial associations and correlations among markers [104]. This oversimplification constrains AI models in cohort‐level analyses to rely solely on the limited set of protein markers directly measured, yielding only coarse annotations of cell or tumor types [105]. It also prevents such models from leveraging marker–marker relationships learned during pretraining to infer the spatial distribution of additional cell subtype markers, cell state markers, or even other genes. As a result, the capacity to transcend the multiplexing limits of spatial proteomics (i.e., the maximum number of detectable genes) remains restricted, hindering the derivation of more refined scientific and clinical insights and ultimately reducing outputs to relatively coarse classifications or diagnostic labels [106]. Furthermore, segmentation‐based strategies often underperform in densely packed tissues, in the presence of imaging artifacts, or in morphologically complex regions, leading to a coexistence of annotation bias and loss of biologically relevant information [107].

#### 5.3.1 KRONOS: A Vision Transformer–Based Foundation Model for Multiplex Histology

The KRONOS model was developed as a foundation model to address these challenges [98]. As a foundation model for histological imaging, KRONOS is built upon a Vision Transformer (ViT‐S/16) [108, 109] backbone augmented with convolutional layers and is pretrained using the DINO‐v2 [110] self‐supervised student–teacher framework, which combines self‐distillation with masked image modeling, to learn generalizable representations from multiplexed spatial proteomics images. The pretraining dataset, SPM‐47 M, comprises over 47 million images collected across diverse tissue sources and imaging platforms. Prior to model training, the dataset underwent manual quality control and artifact masking, and markers with absent or incorrect staining patterns were excluded. Each image was then divided into 256 pixels × 256 pixels patches with 50% overlap to increase diversity. For batch construction, each training instance included one nuclear stain (e.g., DAPI) and two randomly selected protein markers, ensuring both cell localization capability and marker diversity, while all markers were normalized using global mean and variance statistics [111]. Data augmentation strategies, including random cropping, flipping, and rotation, were further applied during training, and a two‐stage stratified sampling strategy was implemented to mitigate bias toward specific tissue–platform combinations [112].

Unlike conventional vision foundation models designed for fixed RGB channels, KRONOS must handle a variable number of marker channels and retain the flexibility to incorporate previously unseen markers during inference. To achieve this, the model introduces a combination of shared convolutional embeddings and marker‐specific encodings. Specifically, the shared convolutional filters capture universal texture patterns and spatial features present across images of different protein markers, eliminating the inefficiency and rigidity of training separate convolutional filters for each marker [113]. At the same time, each marker is assigned a non‐learnable sinusoidal encoding that is both unique across channels and directly extensible to new markers during inference, thereby providing a stable and scalable identification scheme. This combined design ensures that KRONOS can learn efficiently during pretraining while seamlessly generalizing to arbitrary numbers of markers at inference without retraining. For spatial representation, KRONOS further incorporates learnable positional encodings to preserve two‐dimensional coordinates, ensuring that each token embedding reflects not only local image features but also its marker identity and tissue location. After a series of Transformer layers with self‐attention, the model outputs hierarchical contextual embeddings, including global image‐level features (via the CLS token), aggregated marker‐specific features, and cross‐marker location‐specific embeddings. This multi‐level embedding scheme supports both global tissue‐level tasks, such as tissue phenotyping and regional classification, and fine‐grained analyses at the cellular or microregional level. Overall, the architectural innovations of KRONOS lie in (i) the shared convolutional filters for universal feature extraction and (ii) the non‐learnable marker encodings for marker differentiation and scalability, enabling the model to transcend fixed‐channel constraints and serve as a general foundation model for multiplexed spatial proteomics [114].

Given its architecture, methodology, and training dataset, KRONOS demonstrates robust downstream analytical capabilities, including cell‐ and patch‐based phenotyping, region classification and artifact detection, unsupervised tissue phenotyping, multiplex image reverse retrieval in the embedding space, and spatial biomarker discovery with patient stratification. However, KRONOS remains fundamentally an image‐driven model, and its applicability is limited in experimental settings where histological images are unavailable, such as certain label‐free MS‐based spatial proteomics platforms [115, 116].

#### 5.3.2 HEIST: A Hierarchical Graph Transformer for Histology–Expression Integration

With the rapid development of spatial transcriptomics and proteomics, a major challenge has been the construction of unified frameworks that can jointly leverage histological images, molecular expression profiles, and spatial neighborhood information. Existing approaches either fail to explicitly model cell–cell interactions (e.g., scGPT [117], scFoundation [118]) or overlook the role of gene regulatory networks (GRNs) within the spatial microenvironment. To address this gap, the recently proposed HEIST (Histology‐Expression Integration with Spatial Transformers) [99] model offers a graph foundation model‐based strategy.

Architecturally, HEIST is formulated as a hierarchical graph transformer designed to jointly learn cell‐ and gene‐level representations [119]. The model takes two types of graphs as input: a cell graph that encodes spatial interactions between cells, and a gene regulatory network (GRN) for each individual cell that captures intra‐cellular gene–gene interactions [120, 121]. During training, HEIST first performs intra‐level message passing within each graph to extract local structural features, followed by cross‐level message passing to integrate information across the cell and gene hierarchies [122]. In this bidirectional scheme, gene embeddings are updated according to their parent cell's spatial context, while cell embeddings are refined by aggregating the states of their constituent genes [104]. A directional attention mechanism underlies this cross‐level communication, allowing gene representations to be shaped by microenvironmental context and cell representations to embed transcriptional states [123]. Unlike feature concatenation or symmetric attention, this design preserves the natural hierarchy of spatial omics data, maintaining the distinct biological semantics of genes and cells while modeling their interdependence [124]. As a result, HEIST learns spatially informed and biologically grounded embeddings that generalize across tissues and experimental conditions [125].

HEIST's key innovation lies in jointly modeling cell–cell interactions and gene regulatory networks (GRNs) [126], with cross‐level information exchange that bridges gene regulation and tissue phenotypes. Each cell is paired with a GRN derived from co‐expression, while spatial graphs capture neighborhood interactions, allowing microenvironments to shape regulatory states [127]. Through bidirectional updates, gene embeddings integrate cellular context, whereas cell embeddings are refined by their constituent gene features, yielding spatially dependent latent representations [128]. Building on these innovations, HEIST demonstrates state‐of‐the‐art (SOTA) performance across diverse applications—including clinical outcome prediction, cell type annotation, gene imputation, and cell clustering—underscoring its versatility and strength as a foundation model for spatial omics [129].

#### 5.3.3 totalVI: An Autoencoder Framework for Cross‐Modality Integration

With the advent of multimodal single‐cell technologies such as CITE‐seq [115, 116] and their spatial application, such as spatial CITE‐seq [13, 130], it has become possible to measure both transcriptomic and proteomic signals within the same cell. However, these data are characterized by high levels of noise, missing values, and batch effects, making joint analysis prone to bias. Conventional approaches that either analyze each modality separately or concatenate them directly fail to capture the complex dependencies between RNA and protein signals and often underutilize their complementary information.

To overcome these challenges, Gayoso et al. introduced totalVI (total Variational Inference) [131], a deep generative framework based on the variational autoencoder (VAE). totalVI provides a unified probabilistic model for RNA counts and antibody‐derived protein measurements by introducing a shared latent representation that captures biological variation across both modalities, while simultaneously modeling modality‐specific technical noise. In practice, totalVI employs negative binomial likelihoods for transcriptomic data and zero‐inflated negative binomial (ZINB) or related distributions for protein counts, thereby accommodating the differing noise structures inherent to each modality. Through end‐to‐end training, the model performs batch effect correction and yields a harmonized low‐dimensional embedding that integrates both data types [131].

The significance of totalVI lies in its ability to address the fundamental challenge of multimodal data integration while supporting a wide range of downstream analyses, including differential expression testing, missing value imputation, data denoising (outputting batch‐corrected, normalized expression matrices, typically represented in CPM or comparable units), and multimodal joint visualization [18]. Applications in multimodal single‐cell studies have demonstrated that jointly modeling RNA and protein modalities substantially improves the accuracy of cell type annotation and state inference [132]. Nonetheless, certain limitations remain. totalVI is computationally demanding, particularly for large‐scale datasets where GPU acceleration is essential [133]. In addition, as a deep generative model, its interpretability is inherently limited, requiring careful consideration when drawing biological conclusions from features in the latent space [134]. Finally, totalVI was originally designed for single‐cell datasets such as CITE‐seq that measure both RNA and surface proteins; its direct application to purely spatial transcriptomics or to spatial proteomics data derived from mass spectrometry and histological imaging will require further methodological development and adaptation [135].

#### 5.3.4 scArches: A Model‐Surgery Approach for Efficient Integration of New Datasets

A persistent challenge in single‐cell and spatial omics is how to integrate newly generated query datasets with large‐scale reference atlases. Conventional approaches often require joint retraining of the reference and query data, which is computationally expensive and impractical when new datasets are continuously added. Moreover, in cross‐platform, cross‐species, or cross‐modality settings, relying solely on batch correction or alignment strategies often fails to ensure robust generalization and accuracy.

scArches (single‐cell architectural surgery) [131] addresses this challenge by introducing an innovative framework based on architectural modifications of pretrained variational autoencoders (VAEs). The key idea is to perform “surgery” on an existing trained model—such as scVI [136], scANVI [137], trVAE [138] or totalVI [18]—by adding new nodes or parameter modules to the encoder‐decoder architecture while freezing the weights of the pretrained backbone. Only these newly introduced parameters are trained, enabling query data to be embedded into the latent space of the reference model without retraining the entire network. In effect, scArches implements incremental learning by combining “frozen old parameters + trainable new modules,” thereby aligning new datasets with a stable, pretrained latent representation [131].

This architectural strategy effectively resolves the major limitations of traditional integration methods. First, it drastically reduces computational cost, allowing thousands of new samples to be integrated rapidly without retraining the full model. Second, by maintaining the stability of the reference latent space, it prevents representation drift that would otherwise arise from repeated retraining. Third, because the framework is not tied to a specific data modality, scArches can be applied broadly across VAE‐based models, including scVI (transcriptomics), scANVI [137] (semi‐supervised transcriptomics with partial labels), and totalVI (transcriptome plus protein).

The practical utility of scArches is substantial: it supports automated annotation using reference atlases [131], identification of disease‐associated states and novel subpopulations [139], multimodal integration (e.g., CITE‐seq with ATAC or TCR with RNA) [139], imputation of missing modalities (e.g., CITE‐seq protein inference) [140], cross‐batch integration and batch correction [141], mapping of single‐cell data onto spatial references [142], and functional analysis via gene programs [143]. Nonetheless, several limitations should be noted. The method assumes sufficient biological similarity between query and reference datasets; if entirely novel cell types or molecular patterns are present, accurate mapping may be challenging [144]. Additionally, while scArches provides efficient incremental learning, its performance can be hindered by low‐quality or highly noisy data [145]. The key AI models driving advances in spatial proteomics are summarized in Figure 7. KRONOS leverages a Vision Transformer backbone pretrained on 47 million multiplexed images to extract generalizable features and support flexible marker integration. HEIST employs a hierarchical graph transformer architecture that jointly models cell–cell interactions and gene regulatory networks. TotalVI provides an autoencoder‐based framework for cross‐modality integration and batch correction, while scArches enables efficient incremental learning for large‐scale cohort integration. Together, these models represent the current state‐of‐the‐art in AI‐driven spatial proteomics analysis.

In summary, spatial proteomics provides a crucial perspective for understanding tumor heterogeneity and the microenvironment by revealing the in situ distribution of proteins within tissues. Artificial intelligence, particularly deep learning, graph neural networks, and autoencoder‐based models, addresses key challenges of traditional approaches in analyzing complex spatial data, including subjectivity, limited throughput, and difficulties in multimodal integration. Representative technologies such as FLASHDeconv, KRONOS, HEIST, totalVI, and scArches have each advanced data processing and biological discovery from different angles, encompassing mass spectrometry deconvolution, image interpretation, graph‐based modeling, multimodal alignment, and incremental learning. The deep integration of these two domains is propelling precision medicine toward a new era of **“spatial intelligence,”** enabling a closed‐loop translation from data to personalized therapeutic strategies.

Although AI‐driven models substantially enhance the analytical power of spatial proteomics [146, 147], their outputs should not be interpreted in isolation. Effective integration requires iterative feedback between computational predictions and expert pathology review [148, 149]. Pathologists provide critical contextual validation, guiding model refinement, resolving ambiguous phenotypes, and preventing over‐interpretation of purely data‐driven patterns. Future workflows are likely to adopt human–AI collaborative frameworks [150], in which AI performs large‐scale pattern discovery and hypothesis generation, while expert interpretation ensures biological plausibility and clinical relevance.

## 6 Future Perspectives and Challenges

### 6.1 Technological Innovations and Methodological Advancements

Future developments in spatial proteogenomics are expected to achieve breakthroughs across multiple technological dimensions. Foremost among these is the enhancement of spatial resolution, which will serve as a key driving force. Although current technologies have reached single‐cell resolution, precise analysis at the subcellular level remains limited. The integration of next‐generation super‐resolution imaging techniques—such as Expansion Microscopy—with spatial omics holds great promise for achieving nanoscale molecular localization [151]. This convergence will enable researchers to precisely visualize the spatial dynamics of protein distribution, RNA localization, and molecular interactions within organelles, providing unprecedented insights into the spatial organization of intracellular signaling networks in tumor cells.

Advances in multimodal data acquisition technologies will further expand the boundaries of spatial proteogenomics. Future platforms will be capable of simultaneously capturing spatial information on proteins, RNA, DNA, metabolites, and even epigenetic modifications within the same tissue section [152]. For instance, the integration of spatial metabolomics with proteogenomics will uncover the spatial heterogeneity of metabolic reprogramming within the tumor microenvironment, providing critical insights into how tumor cells adapt locally through metabolic plasticity to evade immune surveillance. Such multidimensional molecular atlases will establish a more comprehensive view of tumor biology and drive precision medicine toward deeper and more integrative levels.

The integration of live imaging technologies with spatial omics represents another important direction for future development. Traditional spatial proteogenomic analyses rely on fixed tissue samples and are thus unable to capture dynamic biological processes. Emerging live spatial imaging approaches—such as three‐dimensional tissue imaging based on light‐sheet microscopy and multiphoton microscopy, combined with programmable fluorescent probes—will enable real‐time spatial tracking of molecular events throughout tumor initiation and progression [153]. This dynamic spatial omics paradigm will provide a new spatiotemporal dimension for understanding key processes such as tumor metastasis, angiogenesis, and immune cell infiltration.

Spatial proteogenomics holds tremendous potential for clinical translation, yet it also faces numerous challenges. In the diagnostic domain, spatial omics‐based molecular pathology is poised to redefine cancer classification and grading standards [27]. Traditional pathological diagnosis primarily relies on morphological characteristics, whereas spatial proteogenomics provides spatially resolved molecular information that enables more precise tumor stratification. In future clinical practice, digital pathology platforms are expected to integrate conventional histological staining with spatial molecular profiling, offering pathologists multidimensional diagnostic insights.

### 6.2 Failure Modes and Pitfalls of Multimodal Integration

While multimodal integration promises comprehensive molecular characterization, several failure modes and pitfalls warrant careful consideration before drawing biological conclusions from integrated spatial proteogenomic datasets.

**Conflicting signals across modalities**. A fundamental challenge in multimodal integration is the biological discordance between RNA and protein measurements. RNA–protein correlation varies substantially across genes, with median Spearman correlations typically ranging from 0.4 to 0.6 in bulk tissue studies and often lower in single‐cell analyses [154, 155]. This discordance arises from multiple biological mechanisms: post‐transcriptional regulation, including microRNA‐mediated silencing and RNA‐binding protein interactions; differential mRNA and protein half‐lives spanning minutes to days; translational efficiency variations; and extensive post‐translational modifications that alter protein stability and function [156]. In the tumor microenvironment, such discordance can be particularly pronounced—for example, immune checkpoint proteins like PD‐L1 may show poor correlation with their corresponding transcripts due to rapid protein turnover and post‐translational glycosylation [157]. Similarly, metabolite–enzyme mismatches frequently occur when enzyme abundance does not predict metabolic flux, as allosteric regulation, substrate availability, and compartmentalization govern actual enzymatic activity [158, 159]. Researchers should interpret integrated datasets with awareness that apparent “conflicts” may reflect genuine biological regulation rather than technical artifacts.

**Registration errors, batch effects, and spatial resolution mismatches**. When integrating separately acquired spatial datasets, image registration errors can introduce spurious spatial correlations or mask true biological relationships. Registration accuracy depends on tissue deformation during processing, landmark identification, and algorithmic precision, with typical errors ranging from 10–100 µm depending on the method employed [160]. Batch effects represent another major confounder, arising from differences in tissue processing, staining protocols, instrument calibration, and data acquisition timing [161]. Even within the same platform, day‐to‐day variations can introduce systematic biases that confound biological signals. Spatial resolution mismatches pose additional challenges: integrating 55‐µm Visium spots with single‐cell IMC data requires deconvolution algorithms that make assumptions about cell type composition and spatial mixing, potentially introducing artifacts when these assumptions are violated [32]. The mismatch between sequencing‐based methods (capturing averaged signals from defined areas) and imaging‐based methods (providing single‐cell or subcellular resolution) necessitates careful consideration of the appropriate analytical scale.

**Cases where integration may be misleading or biologically ambiguous**. Several scenarios can lead to misleading conclusions from multimodal integration. First, technical artifacts may masquerade as biological signals—for instance, tissue autofluorescence, antibody cross‐reactivity, or mass spectrometry ion suppression can create apparent spatial patterns unrelated to true molecular distributions [162]. Second, the “ecological fallacy” applies when correlations observed at tissue‐level resolution do not hold at the single‐cell level, leading to incorrect inferences about cell‐autonomous processes [163]. Third, temporal asynchrony between modalities—where RNA reflects recent transcriptional activity while proteins represent accumulated translation over longer timescales—can obscure dynamic biological processes such as cell state transitions or drug responses [164]. Fourth, integration algorithms themselves may introduce biases: variational autoencoders and other deep learning methods can hallucinate patterns in the latent space that lack biological meaning, particularly when training data are limited or unrepresentative [165]. Finally, the absence of ground truth for most integrated analyses makes validation challenging; researchers should employ orthogonal validation strategies, including targeted experiments, independent cohorts, and biological plausibility assessment, before drawing definitive conclusions from computationally integrated spatial proteogenomic data [166].

### 6.3 Challenges in Clinical Translation

The translation of spatial proteogenomics into routine clinical practice faces several quantifiable technical and practical challenges that must be addressed [5, 167, 168]. Current spatial proteomics technologies operate across a wide resolution spectrum [169]. Antibody‐based methods such as CODEX and MIBI achieve subcellular resolution (∼300 nm and ∼260 nm, respectively) [51, 52, 170], while mass spectrometry‐based approaches typically operate at 10–100 µm [72]. For reference, a typical mammalian cell measures 10–30 µm in diameter, meaning that only the highest‐resolution platforms can reliably distinguish subcellular protein distributions [169]. Moreover, protein detection depth varies substantially: IMC and MIBI detect 40–50 pre‐selected markers, whereas DVP can quantify ∼1700 proteins per cell but at lower throughput [47]. Future clinical applications will require careful matching of technological capabilities to specific diagnostic needs [54]. A critical practical barrier is the inconsistency between fresh‐frozen and formalin‐fixed paraffin‐embedded (FFPE) tissue processing. While FFPE tissues are the clinical standard due to their long‐term stability and widespread availability in pathology archives, formalin fixation introduces protein crosslinks that can reduce antigen retrieval efficiency by 30%–50% compared to fresh tissue [171]. Different antigen retrieval protocols (heat‐induced vs. enzymatic) further contribute to inter‐laboratory variability. Standardized protocols that account for fixation time, paraffin embedding conditions, and storage duration are urgently needed [172]. The estimated cost per sample for advanced spatial proteomics platforms ranges from $500–$2000 USD for antibody‐based methods (IMC, CODEX) to $3000–$10 000 USD for comprehensive mass spectrometry‐based approaches (DVP, LCM‐NanoPOTS), excluding equipment and personnel costs [173, 174]. Processing time varies from 1–2 days for multiplexed imaging to 1–2 weeks for high‐depth mass spectrometry analyses [47, 72]. These factors currently limit spatial proteogenomics to research settings and high‐value clinical decisions (e.g., immunotherapy selection), though costs are expected to decrease with technological maturation and increased adoption. Beyond technical considerations, spatial proteogenomic data must interface with existing electronic health record (EHR) systems, pathology information systems (LIS), and clinical decision support tools [175]. The large data volumes generated (often gigabytes per sample) and the need for specialized computational infrastructure pose logistical challenges for many clinical laboratories.

Prediction of therapeutic response, including but not limited to cancer therapeutics, is one of the most promising clinical applications of spatial proteogenomics. By analyzing the spatial molecular features of pretreatment tumor samples, it is possible to predict patient responses to specific therapeutic regimens [176]. For example, the efficacy of immune checkpoint inhibitors is closely associated with the spatial organization of the tumor immune microenvironment [177]. Spatial proteogenomics enables precise characterization of the spatial distribution and activation states of immune cells, as well as their interaction patterns with tumor cells, thereby facilitating the identification of patient subgroups most likely to benefit from immunotherapy.

However, major challenges to clinical translation include the establishment of standardized workflows, cost‐effectiveness evaluation, and regulatory approval. At present, technical variations across platforms and laboratories result in poor data comparability, underscoring the urgent need for unified technical standards and quality control systems [168]. International initiatives such as the Human Cell Atlas and The Cancer Genome Atlas are actively working to develop standardized guidelines for spatial omics data, yet this process will require broad international collaboration and consensus.

### 6.4 Future Research Directions and Technological Convergence

In the future, spatial proteogenomics will increasingly converge with other frontier technologies, opening new avenues of research. The application of quantum computing is expected to overcome current computational bottlenecks, particularly in handling high‐dimensional spatial data and simulating complex molecular interactions. Quantum algorithms may bring revolutionary breakthroughs in areas such as protein folding prediction and drug‐target interaction modeling, directly benefiting drug discovery processes based on spatial proteogenomic insights.

The integration of synthetic biology with spatial omics will establish an entirely new research paradigm. By designing programmable molecular probes and reporter systems, researchers can dynamically monitor the spatial distribution of specific molecular events within living tissues. For instance, spatial barcoding technologies developed using the CRISPR–Cas system enable the genomic recording of cellular spatial locations and developmental lineages, providing a powerful tool for elucidating tumor clonal evolution and metastasis.

The convergence of organoid and organ‐on‐a‐chip technologies with spatial proteogenomics will advance the development of in vitro disease models. These three‐dimensional culture systems can partially recapitulate tissue architecture and cell–cell interactions. When combined with spatial omics analysis, they allow investigation of the spatial dynamics of tumor initiation and progression under controlled experimental conditions. This approach not only reduces dependence on clinical specimens but also enables high‐throughput drug screening and validation of personalized therapeutic strategies.

The application of nanotechnology will further enhance the capabilities of spatial proteogenomics. Nanoscale probes and sensors can achieve single‐molecule detection and localization, providing unprecedented spatial resolution. Moreover, nanocarrier systems can precisely deliver therapeutic agents to specific spatial regions within tumors, enabling truly spatially guided precision therapy.

## 7 Conclusion

The emergence of spatial proteogenomics marks the beginning of a new era in cancer research. This revolutionary technology transcends the limitations of traditional methods by seamlessly integrating molecular information with spatial context, providing an unprecedented perspective for deciphering the complexity of tumor biology. From bulk tissue analyses to single‐cell sequencing and now to integrated spatial multi‐omics, each technological leap has deepened our understanding of the fundamental nature of cancer. Spatial proteogenomics is not only the inevitable outcome of technological evolution but also a pivotal driving force in the advancement of precision medicine.

As reviewed in this article, spatial proteogenomics demonstrates transformative impact across multiple dimensions. At the level of basic research, it has revealed finely tuned regulatory networks of cell–cell interactions within the tumor microenvironment, elucidated the spatial organization underlying tumor heterogeneity, and identified novel neoantigens from the dark proteome that are undetectable by conventional methods. In clinical applications, this technology is reshaping the paradigms of cancer diagnosis, prognostic evaluation, and therapeutic decision‐making. Through the integration of artificial intelligence and machine learning algorithms, the clinical value of spatial proteogenomic data is being fully realized, providing a robust molecular foundation for personalized medicine.

However, it is essential to recognize that spatial proteogenomics remains in its early stages of development. Numerous challenges—including technical standardization, data integration, and clinical validation—must be systematically addressed. Interdisciplinary collaboration will be key to sustaining progress in this field. Biologists, computational scientists, clinicians, engineers, and data scientists must work closely together to build a complete translational pipeline from basic research to clinical application. At the same time, policymakers, ethicists, and the broader society should also be engaged to ensure that technological advancements ultimately benefit all of humanity.

Looking ahead, spatial proteogenomics will continue to drive a paradigm shift in cancer research and therapy. As technology matures and costs gradually decrease, this approach is expected to become a routine tool in cancer diagnosis and treatment. More importantly, the integrative and systematic mindset embodied by spatial proteogenomics will shape the broader trajectory of biomedical research. It reminds us that the complexity of life arises not only from molecular diversity but also from the exquisite spatiotemporal organization of biological systems.

As stated at the beginning of this review, spatial proteogenomics can be regarded as the “GPS of biology,” guiding us to navigate precisely through the intricate molecular landscape. This analogy not only captures the functional essence of technology but also underscores its profound significance, offers direction for patients lost in the labyrinth of cancer, and paves the way toward the realization of precision medicine. With continued technological advances and expanding clinical applications, there is every reason to believe that spatial proteogenomics will play an increasingly vital role in overcoming one of humanity's greatest health challenges, ultimately achieving truly personalized precision medicine and improving both patient survival and quality of life.

## Author Contributions

Yan Zhang and Joe Yeong conceptualized the review. Yida Wang, Yang Wu, Feng Zhang, Parthiban Periasamy, Haiyue You, Denise Goh, Rachel Elizabeth Ann Fincham, Xin Ning, Danping Wu, Lu Liu, Ying Jiang and Zhiwen Qian wrote the manuscript. Yida Wang, Yang Wu, Rachel Elizabeth Ann Fincham, Feng Zhang and Haiyue You prepared the figures and tables. Yan Zhang and Joe Yeong critically reviewed and edited the manuscript. Yan Zhang and Joe Yeong got funding support. All authors read and approved of the final manuscript.

## Funding

The work was supported by the National Natural Science Foundation of China (82472842 and 82473350), Wuxi Double‐Hundred Talent Fund Project (BJ2023075), Singapore National Medical Research Council (OFLCG23may‐0039, OFLCG24MAY‐0028, OFLCG24may‐0025), A*STAR gap funding (I23D1AG121, I24D1AG059, I24D1AG082) and A*STAR non‐core funding (SC15/19‐301111‐RSC‐OOE).

## Ethics Approval and Consent to Participate

This study was approved by the Institutional Review Board of Agency for Science, Technology and Research (A*STAR), Singapore (IRB approval ID: 2021–188). This article does not contain any studies with human participants or animals performed by any of the authors, thus written consent is not applicable.

## Conflicts of Interest

The authors declare no conflicts of interest.

## Supporting information




**Supporting File**: advs74321‐sup‐0001‐SuppMat.docx.

## Data Availability

Data and materials can be provided at reasonable request to the corresponding author.
