# Supplementary material for: Integrating Spatial Proteogenomics in Cancer Research
Source: Adv Sci (Weinh). 2026 Feb 8;13(29):e20744. doi: 10.1002/advs.202520744 (PMC13205702; doi:10.1002/advs.202520744)
Supplement: Supplementary file 1 — Supporting File: advs74321‐sup‐0001‐SuppMat.docx. [file ADVS-13-e20744-s001.docx]

Table S1.Evolutionary Timeline of Spatial Proteogenomic Technologies

| Category | Year | Technology | Key Innovation | | Problem Solved  (vs. Previous Generation) | Resolution | Markers/Detection depth | Original Paper Citation |
| --- | --- | --- | --- | --- | --- | --- | --- | --- |
| Spatial Multi-Omics with One Platform | 1997 | MALDI-MSI | Matrix-assisted laser desorption imaging | Label-free spatial mapping (vs. IHC: antibody-dependent) | | ~50–200 μm | Metabolome +Lipidome + Limited Proteome | ^1^ |
|  | 2017 | CITE-seq | Antibody-DNA tags for single-cell | Simultaneous RNA + protein (vs. separate scRNA-seq and FACS) | | Single-cell | RNA + ~200 proteins | ^2^ |
|  | 2020 | DBiT-seq | Deterministic barcoding in tissue | First spatial RNA + protein co-detection (vs. CITE-seq: no spatial) | | 10 μm | Transcriptome + ~30 proteins | ^3^ |
|  | 2022 | SM-Omics | Spatial multi-omics on TMAs | High-throughput on tissue arrays (vs. DBiT-seq: single section) | | 55 μm | Transcriptome + proteins | ^4^ |
|  | 2023 | SPOTS | Protein staining + spatial transcriptomics | Simpler workflow (vs. DBiT-seq: complex protocol) | | 55 μm | Transcriptome + proteins | ^5^ |
|  | 2023 | Spatial CITE-seq | High-plex ADT + spatial barcoding | Expanded protein panel (vs. DBiT-seq: ~30 proteins) | | 10 μm | Transcriptome + ~200 proteins | ^6^ |
|  | 2023 | Spatial epigenome-transcriptome | Chromatin + RNA co-profiling | Added epigenetic layer (vs. RNA only) | | 20 μm | ATAC + RNA | ^7^ |
|  | 2024 | Spatial Tri-omics | Chromatin + RNA + protein | Three modalities in one section (vs. dual-omics) | | 20 μm | ATAC + RNA + protein | ^8^ |
|  | 2024 | spatial-Mux-seq | RNA + protein + chromatin + mito + lipid (Five omics modalities) | Comprehensive molecular portrait (vs. tri-omics: three layers) | | Variable | Five modalities | ^9^ |
|  | 2025 | OmiCLIP | H&E to transcriptome prediction | Bridge histology and omics without spatial assay (vs. spatial-seq: expensive) | | Tissue patch | Predicted transcriptome | ^10^ |
| Spatial Transcriptomics | 2016 | ST (Spatial Transcriptomics) | Spatially barcoded capture arrays | Enabled transcriptome-wide spatial mapping (vs. FISH: ~10 genes) | | ~100 μm | Whole transcriptome | ^11^ |
|  | 2019 | Slide-seq | DNA-barcoded beads on glass | Improved resolution 10-fold (vs. ST: 100 μm) | | ~10 μm | Whole transcriptome | ^12^ |
|  | 2020 | 10x Visium | Commercialized ST platform | Standardization and accessibility (vs. ST: lab-specific) | | 55 μm | Whole transcriptome + H&E from same section | ^13^ |
|  | 2021 | Slide-seq V2 | Optimized bead loading and RNA capture | Increased sensitivity ~10-fold (vs. Slide-seq: low capture) | | ~10 μm | Whole transcriptome | ^14^ |
|  | 2022 | Stereo-seq | DNA nanoball patterning | Achieved subcellular resolution (vs. Visium: cellular-level) | | 500 nm | Whole transcriptome + ssDNA staining image + H&E | ^15^ |
|  | 2022 | MERSCOPE | MERFISH-based imaging | True single-cell in situ detection with high sensitivity (vs. Slide-seq: lower efficiency) | | Subcellular | Up to 1,000 genes | ^16^ |
|  | 2023 | Xenium | In situ targeted panel with rapid turnaround | Single-molecule sensitivity in hours (vs. sequencing: days) | | Subcellular | 100-5,000 genes | ^17^ |
|  | 2023 | CosMx SMI | Single-molecule imaging | Simultaneous RNA + protein in situ (vs. Xenium: RNA only initially) | | Subcellular | 1,000 RNA + 100 proteins | ^18^ |
|  | 2024 | Visium HD | 2 μm bin size capture | Single-cell scale resolution with whole transcriptome (vs. Visium: 55 μm) | | 2 μm | Whole transcriptome | ^19^ |
|  | 2024 | MERSCOPE Ultra | MERFISH 2.0 chemistry | Enhanced sensitivity and speed (vs. MERSCOPE: slower acquisition) | | Subcellular | Up to 1,000 genes | ^16^ |
|  | 2024 | Xenium Prime 5K | Expanded gene panel | 5× more targets per run (vs. Xenium: ~1,000 genes) | | Subcellular | 5,000 genes | ^16^ |
|  | 2025 | Xenium Protein | RNA + Protein co-detection | Same-section spatial multiomics (vs. Xenium: RNA only) | | Subcellular | RNA + protein | ^11^ |
| Antibody-Based Spatial Proteomics | 2014 | IMC (Imaging Mass Cytometry) | Metal-tagged antibodies + laser ablation | Overcame fluorescence spectral overlap (vs. IF: ~4-5 markers) | | ~1 μm | ~40 proteins | ^20^ |
|  | 2014 | MIBI | Secondary ion mass spectrometry | Higher spatial resolution (vs. IMC: ~1 μm) | | ~260 nm | ~40 proteins | ^21^ |
|  | 2018 | CODEX | DNA-barcoded antibodies + iterative imaging | Expanded markers without mass spec (vs. IMC/MIBI: expensive) | | ~300 nm | ~60 proteins | ^22^ |
|  | 2019 | GeoMx DSP | UV-cleavable barcodes + digital counting | ROI-based flexible profiling (vs. CODEX: whole-tissue imaging) | | 10-600 μm (ROI) | Up to 1,200 proteins | ^20^ |
|  | 2020 | IBEX | Iterative bleaching + multiplexed staining | Unlimited marker expansion via cycling (vs. CODEX: fixed panel) | | ~300 nm | >65 proteins | ^23^ |
|  | 2022 | PhenoCycler (CODEX) | Commercial platform with automation | Standardized workflow (vs. CODEX: manual protocol variability) | | ~300 nm | ~100 proteins | ^24^ |
|  | 2023 | COMET | Combinatorial epitope imaging | Reduced antibody consumption (vs. CODEX: one Ab per marker) | | Subcellular | >100 proteins | ^22^ |
|  | 2024 | PhenoCycler-Fusion 2.0 | Enhanced automation and speed | Higher throughput (vs. PhenoCycler: slower acquisition) | | ~300 nm | >100 proteins | ^24^ |
|  | 2024 | MACSima | Fully automated cyclic IF | Walk-away automation (vs. CODEX/IBEX: manual intervention) | | Subcellular | >100 proteins | ^25^ |
| MS-Based Spatial Proteomics | 2018 | nanoPOTS | Nanoliter sample processing | Enabled analysis from ~10–100 cells (vs. bulk MS: thousands) | | ~100 μm | ~3,000 proteins | ^26^ |
|  | 2018 | LCM-nanoPOTS | Laser capture microdissection + nanoPOTS | Precise spatial targeting (vs. nanoPOTS: manual isolation) | | 20–100 μm | ~3,000 proteins | ^27^ |
|  | 2020 | LCM-nanoPOTS v2 | Optimized sensitivity | 20× more proteins detected (vs. LCM-nanoPOTS v1: ~100 proteins) | | 20–100 μm | >2,000 proteins | ^28^ |
|  | 2022 | DVP (Deep Visual Proteomics) | AI-guided segmentation + microdissection | Phenotype-specific cell isolation (vs. LCM: morphology-based) | | Single-cell | ~1,700 proteins/cell | ^29^ |
|  | 2022 | PiMS | Top-down proteoform imaging | Preserved PTMs and proteoforms (vs. BUP: lost in digestion) | | ~50 μm | Intact proteoforms | ^30^ |
|  | 2023 | Auto-PiMS | Automated proteoform imaging | Increased throughput (vs. PiMS: manual operation) | | ~50 μm | Intact proteoforms | ^31^ |
|  | 2023 | LCM-nanoPOTS v3 | Further optimized for islets | >6,000 proteins from small tissue (vs. v2: ~2,000) | | 10 μm | >6,000 proteins | ^26^ |
|  | 2025 | wcSOP | Wet collection + surfactant one-pot | Simplified workflow, lower cost (vs. LCM-nanoPOTS: specialized chips) | | 20–100 μm | >4,000 proteins | ^32^ |
|  | 2025 | DVP 2.0 | Enhanced single-cell depth | Proteotoxicity mapping in disease (vs. DVP: limited clinical) | | Single-cell | >2,000 proteins/cell | ^33^ |
| Traditional ML- and AI-based Proteogenomics Methods | 2018 | scVI | Deep generative model for scRNA-seq | Probabilistic latent space (vs. PCA: linear only) | | — | Transcriptomics | ^34^ |
|  | 2020 | TotalVI | VAE for CITE-seq integration | Joint RNA-protein modeling (vs. separate analysis) | | — | RNA + protein | ^35^ |
|  | 2020 | FLASHDeconv | Ultrafast MS deconvolution | Faster TDP analysis (vs. ProSightPC: slow) | | — | MS spectra | ^36^ |
|  | 2021 | Cell2location | Probabilistic cell type deconvolution | Reference-based spatial mapping (vs. clustering: no annotation) | | — | Spatial deconvolution | ^37^ |
|  | 2021 | Tangram | Deep learning for spatial mapping | Single-cell to spatial alignment (vs. correlation: low accuracy) | | — | scRNA-seq mapping | ^38^ |
|  | 2022 | scArches | Transfer learning architecture | Efficient model updating (vs. de novo: computationally expensive) | | — | Reference mapping | ^39^ |
|  | 2024 | scGPT | Transformer-based foundation model | Generalizable across tasks (vs. task-specific models) | | — | Multi-task | ^40^ |
|  | 2024 | scFoundation | Large-scale pretraining | Learned from 50M+ cells (vs. scGPT: smaller dataset) | | — | Foundation model | ^41^ |
|  | 2025 | HEIST | Hierarchical graph transformer | Joint cell + GRN modeling (vs. GNNs: single scale) | | — | Spatial + GRN | ^42^ |
|  | 2025 | KRONOS | Foundation model for spatial proteomics | Generalizable from 47M images (vs. task-specific: limited transfer) | | — | Flexible markers | ^43^ |
|  | 2025 | OmiCLIP | Visual-omics foundation model | H&E to spatial transcriptome prediction (vs. paired data: expensive) | | — | Histology-omics | ^10^ |
|  | 2025 | scGPT-spatial | Spatial extension of scGPT | Continual pretraining for spatial data (vs. scGPT: non-spatial) | | — | Spatial transcriptomics | ^44^ |

References

1. Caprioli RM, Farmer TB, Gile J. Molecular imaging of biological samples: Localization of peptides and proteins using MALDI-TOF MS. *Anal Chem*. 1997;69(23):4751-4760. doi:10.1021/ac970888i

2. Stoeckius M, Hafemeister C, Stephenson W, et al. Simultaneous epitope and transcriptome measurement in single cells. *Nat Methods*. 2017;14(9):865-868. doi:10.1038/nmeth.4380

3. Liu Y, Yang M, Deng Y, et al. High-spatial-resolution multi-omics sequencing via deterministic barcoding in tissue. *Cell*. 2020;183(6):1665-1681.e18. doi:10.1016/j.cell.2020.10.026

4. Vickovic S, Lötstedt B, Klughammer J, et al. SM-omics is an automated platform for high-throughput spatial multi-omics. *Nat Commun*. 2022;13(1):795. doi:10.1038/s41467-022-28445-y

5. Ben-Chetrit N, Niu X, Swett AD, et al. Integration of whole transcriptome spatial profiling with protein markers. *Nat Biotechnol*. 2023;41(6):788-793. doi:10.1038/s41587-022-01536-3

6. Liu Y, DiStasio M, Su G, et al. High-plex protein and whole transcriptome co-mapping at cellular resolution with spatial CITE-seq. *Nat Biotechnol*. 2023;41(10):1405-1409. doi:10.1038/s41587-023-01676-0

7. Zhang D, Deng Y, Kukanja P, et al. Spatial epigenome-transcriptome co-profiling of mammalian tissues. *Nature*. 2023;616(7955):113-122. doi:10.1038/s41586-023-05795-1

8. Zhang D, Rubio Rodríguez-Kirby LA, Lin Y, et al. Spatial dynamics of brain development and neuroinflammation. *Nature*. 2025;647(8088):213-227. doi:10.1038/s41586-025-09663-y

9. Guo P, Mao L, Chen Y, et al. Multiplexed spatial mapping of chromatin features, transcriptome and proteins in tissues. *Nat Methods*. 2025;22(3):520-529. doi:10.1038/s41592-024-02576-0

10. Chen W, Zhang P, Tran TN, et al. A visual-omics foundation model to bridge histopathology with spatial transcriptomics. *Nat Methods*. 2025;22(7):1568-1582. doi:10.1038/s41592-025-02707-1

11. Ståhl PL, Salmén F, Vickovic S, et al. Visualization and analysis of gene expression in tissue sections by spatial transcriptomics. *Science*. 2016;353(6294):78-82. doi:10.1126/science.aaf2403

12. Rodriques SG, Stickels RR, Goeva A, et al. Slide-seq: A scalable technology for measuring genome-wide expression at high spatial resolution. *Science*. 2019;363(6434):1463-1467. doi:10.1126/science.aaw1219

13. Asp M, Giacomello S, Larsson L, et al. A spatiotemporal organ-wide gene expression and cell atlas of the developing human heart. *Cell*. 2019;179(7):1647-1660.e19. doi:10.1016/j.cell.2019.11.025

14. Stickels RR, Murray E, Kumar P, et al. Highly sensitive spatial transcriptomics at near-cellular resolution with slide-seqV2. *Nat Biotechnol*. 2021;39(3):313-319. doi:10.1038/s41587-020-0739-1

15. Chen A, Liao S, Cheng M, et al. Spatiotemporal transcriptomic atlas of mouse organogenesis using DNA nanoball-patterned arrays. *Cell*. 2022;185(10):1777-1792.e21. doi:10.1016/j.cell.2022.04.003

16. Xia C, Fan J, Emanuel G, Hao J, Zhuang X. Spatial transcriptome profiling by MERFISH reveals subcellular RNA compartmentalization and cell cycle-dependent gene expression. *Proc Natl Acad Sci U S A*. 2019;116(39):19490-19499. doi:10.1073/pnas.1912459116

17. Janesick A, Shelansky R, Gottscho AD, et al. High resolution mapping of the tumor microenvironment using integrated single-cell, spatial and in situ analysis. *Nat Commun*. 2023;14(1):8353. doi:10.1038/s41467-023-43458-x

18. He S, Bhatt R, Brown C, et al. High-plex imaging of RNA and proteins at subcellular resolution in fixed tissue by spatial molecular imaging. *Nat Biotechnol*. 2022;40(12):1794-1806. doi:10.1038/s41587-022-01483-z

19. Vickovic S, Eraslan G, Salmén F, et al. High-definition spatial transcriptomics for in situ tissue profiling. *Nat Methods*. 2019;16(10):987-990. doi:10.1038/s41592-019-0548-y

20. Giesen C, Wang HAO, Schapiro D, et al. Highly multiplexed imaging of tumor tissues with subcellular resolution by mass cytometry. *Nat Methods*. 2014;11(4):417-422. doi:10.1038/nmeth.2869

21. Angelo M, Bendall SC, Finck R, et al. Multiplexed ion beam imaging of human breast tumors. *Nat Med*. 2014;20(4):436-442. doi:10.1038/nm.3488

22. Goltsev Y, Samusik N, Kennedy-Darling J, et al. Deep profiling of mouse splenic architecture with CODEX multiplexed imaging. *Cell*. 2018;174(4):968-981.e15. doi:10.1016/j.cell.2018.07.010

23. Radtke AJ, Kandov E, Lowekamp B, et al. IBEX: A versatile multiplex optical imaging approach for deep phenotyping and spatial analysis of cells in complex tissues. *Proc Natl Acad Sci U S A*. 2020;117(52):33455-33465. doi:10.1073/pnas.2018488117

24. Black S, Phillips D, Hickey JW, et al. CODEX multiplexed tissue imaging with DNA-conjugated antibodies. *Nat Protoc*. 2021;16(8):3802-3835. doi:10.1038/s41596-021-00556-8

25. Lin JR, Izar B, Wang S, et al. Highly multiplexed immunofluorescence imaging of human tissues and tumors using t-CyCIF and conventional optical microscopes. *Elife*. 2018;7:e31657. doi:10.7554/eLife.31657

26. Zhu Y, Piehowski PD, Zhao R, et al. Nanodroplet processing platform for deep and quantitative proteome profiling of 10-100 mammalian cells. *Nat Commun*. 2018;9(1):882. doi:10.1038/s41467-018-03367-w

27. Zhu Y, Clair G, Chrisler WB, et al. Proteomic analysis of single mammalian cells enabled by microfluidic nanodroplet sample preparation and ultrasensitive NanoLC-MS. *Angew Chem Int Ed Engl*. 2018;57(38):12370-12374. doi:10.1002/anie.201802843

28. Cong Y, Liang Y, Motamedchaboki K, et al. Improved single-cell proteome coverage using narrow-bore packed NanoLC columns and ultrasensitive mass spectrometry. *Anal Chem*. 2020;92(3):2665-2671. doi:10.1021/acs.analchem.9b04631

29. Mund A, Coscia F, Kriston A, et al. Deep visual proteomics defines single-cell identity and heterogeneity. *Nat Biotechnol*. 2022;40(8):1231-1240. doi:10.1038/s41587-022-01302-5

30. Brunner AD, Thielert M, Vasilopoulou C, et al. Ultra-high sensitivity mass spectrometry quantifies single-cell proteome changes upon perturbation. *Mol Syst Biol*. 2022;18(3):e10798. doi:10.15252/msb.202110798

31. Jp M, P S, Kr D, et al. Automated imaging and identification of proteoforms directly from ovarian cancer tissue. *Nature communications*. 2023;14(1). doi:10.1038/s41467-023-42208-3

32. Kitata RB, Velickovic M, Xu Z, et al. Robust collection and processing for label-free single voxel proteomics. *Nat Commun*. 2025;16(1):547. doi:10.1038/s41467-024-54643-x

33. Rosenberger FA, Mädler SC, Thorhauge KH, et al. Deep visual proteomics maps proteotoxicity in a genetic liver disease. *Nature*. 2025;642(8067):484-491. doi:10.1038/s41586-025-08885-4

34. Lopez R, Regier J, Cole MB, Jordan MI, Yosef N. Deep generative modeling for single-cell transcriptomics. *Nat Methods*. 2018;15(12):1053-1058. doi:10.1038/s41592-018-0229-2

35. Gayoso A, Steier Z, Lopez R, et al. Joint probabilistic modeling of single-cell multi-omic data with totalVI. *Nat Methods*. 2021;18(3):272-282. doi:10.1038/s41592-020-01050-x

36. Jeong K, Kim J, Gaikwad M, et al. FLASHDeconv: Ultrafast, high-quality feature deconvolution for top-down proteomics. *Cell Syst*. 2020;10(2):213-218.e6. doi:10.1016/j.cels.2020.01.003

37. Kleshchevnikov V, Shmatko A, Dann E, et al. Cell2location maps fine-grained cell types in spatial transcriptomics. *Nat Biotechnol*. 2022;40(5):661-671. doi:10.1038/s41587-021-01139-4

38. Biancalani T, Scalia G, Buffoni L, et al. Deep learning and alignment of spatially resolved single-cell transcriptomes with tangram. *Nat Methods*. 2021;18(11):1352-1362. doi:10.1038/s41592-021-01264-7

39. Lotfollahi M, Naghipourfar M, Luecken MD, et al. Mapping single-cell data to reference atlases by transfer learning. *Nat Biotechnol*. 2022;40(1):121-130. doi:10.1038/s41587-021-01001-7

40. Cui H, Wang C, Maan H, et al. scGPT: Toward building a foundation model for single-cell multi-omics using generative AI. *Nat Methods*. 2024;21(8):1470-1480. doi:10.1038/s41592-024-02201-0

41. Hao M, Gong J, Zeng X, et al. Large-scale foundation model on single-cell transcriptomics. *Nat Methods*. 2024;21(8):1481-1491. doi:10.1038/s41592-024-02305-7

42. Madhu H, Rocha JF, Huang T, Viswanath S, Krishnaswamy S, Ying R. HEIST: A graph foundation model for spatial transcriptomics and proteomics data. *ArXiv*. Published online September 25, 2025:arXiv:2506.11152v2.

43. Shaban M, Chang Y, Qiu H, et al. A foundation model for spatial proteomics. *arXiv*. Preprint posted online June 3, 2025:arXiv:2506.03373. doi:10.48550/arXiv.2506.03373

44. See JE, Barlow S, Arjumand W, DuBose H, Segato Dezem F, Plummer J. Spatial omics: Applications and utility in profiling the tumor microenvironment. *Cancer Metastasis Rev*. 2025;44(4):87. doi:10.1007/s10555-025-10304-z
